# BiomarCaRE: rationale and design of the European BiomarCaRE project including 300,000 participants from 13 European countries

**DOI:** 10.1007/s10654-014-9952-x

**Published:** 2014-09-20

**Authors:** Tanja Zeller, Maria Hughes, Tarja Tuovinen, Arne Schillert, Annette Conrads-Frank, Hester den Ruijter, Renate B. Schnabel, Frank Kee, Veikko Salomaa, Uwe Siebert, Barbara Thorand, Andreas Ziegler, Heico Breek, Gerard Pasterkamp, Kari Kuulasmaa, Wolfgang Koenig, Stefan Blankenberg

**Affiliations:** 1 Clinic for General and Interventional Cardiology, University Heart Centre Hamburg, Martinistr. 52, 20246 Hamburg, Germany; 2 German Centre for Cardiovascular Research (DZHK), Partner Site Hamburg/Lübeck/Kiel, Hamburg, Germany; 3 UK Clinical Research Collaboration Centre of Excellence for Public Health, Queens University of Belfast, Belfast, Northern Ireland, UK; 4 National Institute for Health and Welfare, Helsinki, Finland; 5 Institut für Medizinische Biometrie und Statistik, Universität zu Lübeck, Universitätsklinikum Schleswig-Holstein, Campus Lübeck, Lübeck, Germany; 6 Department of Public Health and Health Technology Assessment, UMIT - University for Health Sciences, Medical Informatics and Technology, Hall i.T., Austria; 7 Universitair Medisch Centrum, Utrecht, The Netherlands; 8 Harvard School of Public Health, Harvard Medical School, Boston, MA USA; 9 Institute of Epidemiology II, Helmholtz Zentrum München, German Research Center for Environmental Health, Neuherberg, Germany; 10 German Centers for Cardiovascular Research (DZHK), Partner Site Munich, Munich, Germany; 11 Zentrum für Klinische Studien Lübeck, Universität zu Lübeck, Lübeck, Germany; 12 Cavadis B.V., Utrecht, The Netherlands; 13 Department of Internal Medicine II-Cardiology, University of Ulm Medical Centre, Ulm, Germany

**Keywords:** BiomarCaRE, Biomarker, Cardiovascular Risk Assessment, MORGAM, EU

## Abstract

Biomarkers are considered as tools to enhance cardiovascular risk estimation. However, the value of biomarkers on risk estimation beyond European risk scores, their comparative impact among different European regions and their role towards personalised medicine remains uncertain. Biomarker for Cardiovascular Risk Assessment in Europe (BiomarCaRE) is an European collaborative research project with the primary objective to assess the value of established and emerging biomarkers for cardiovascular risk prediction. BiomarCaRE integrates clinical and epidemiological biomarker research and commercial enterprises throughout Europe to combine innovation in biomarker discovery for cardiovascular disease prediction with consecutive validation of biomarker effectiveness in large, well-defined primary and secondary prevention cohorts including over 300,000 participants from 13 European countries. Results from this study will contribute to improved cardiovascular risk prediction across different European populations. The present publication describes the rationale and design of the BiomarCaRE project.

**Electronic supplementary material:**

The online version of this article (doi:10.1007/s10654-014-9952-x) contains supplementary material, which is available to authorized users.

## Introduction

Despite significant advances in treatment, cardiovascular disease (CVD) remains the leading cause of death worldwide. Traditional risk factors including dyslipidemia, hypertension, diabetes, obesity, and smoking account for a large proportion of the risk of myocardial infarction and stroke globally [36]. Most of these traditional risk factors are modifiable and their management is likely to reduce risk of CVD in both primary and secondary prevention settings [12]. However, risk estimates based on these classical risk factors only partially explain CVD incidence in the general population and risk estimates vary across European populations. Despite the availability of various global risk assessment scores like the Framingham Score [7], the PROCAM Score [3] and the European Society of Cardiology SCORE [6], prediction of cardiovascular events is incomplete and a considerable number of patients at risk go unidentified on the basis of traditional risk factors alone. Khot et al. [18] have reported that 62 % of patients with MI present with none or only one risk factor and less than 10 % show three or four risk factors. To improve risk estimation above and beyond traditional risk scores, and to improve therapy decision making and guidance, novel (emerging) biomarkers are of considerable interest [4]. A number of those biomarkers are thought to provide additional prognostic information; however, only few studies have evaluated a large panel of biomarkers [4]. Therefore, it is not clear whether these proposed biomarkers might merely be proxies for known risk factors and therefore whether they really provide incremental predictive and discriminatory information. Most importantly, are there so far undetected novel biomarkers that might perform even better than known biomarkers?

Molecular omics-based approaches such as profiling of microRNAs, the proteome of microparticles, the transcriptome and the metabolome represent new and promising approaches for the discovery of novel biomarkers and might lead to the identification of markers which better distinguish individuals who may experience incident cardiovascular events. Overall, risk estimation needs to be assessed across populations in different European regions, as well as in the secondary prevention setting where even less is known about the performance of established and novel biomarkers for risk estimation. Ultimately, we need to know whether a modified risk estimation model will translate into a change in treatment [4].

Within the BiomarCaRE project, promising novel biomarkers will be selected in order to develop risk estimation models for incident CVD and they will be validated across European populations. Success in building a robust prediction score could greatly facilitate individualized risk estimation, screening, and diagnosis of CVD. The novel risk score would have the potential to change existing clinical guidelines and could thus have significant public health impact.

### The consortium

**Fig. 1:**
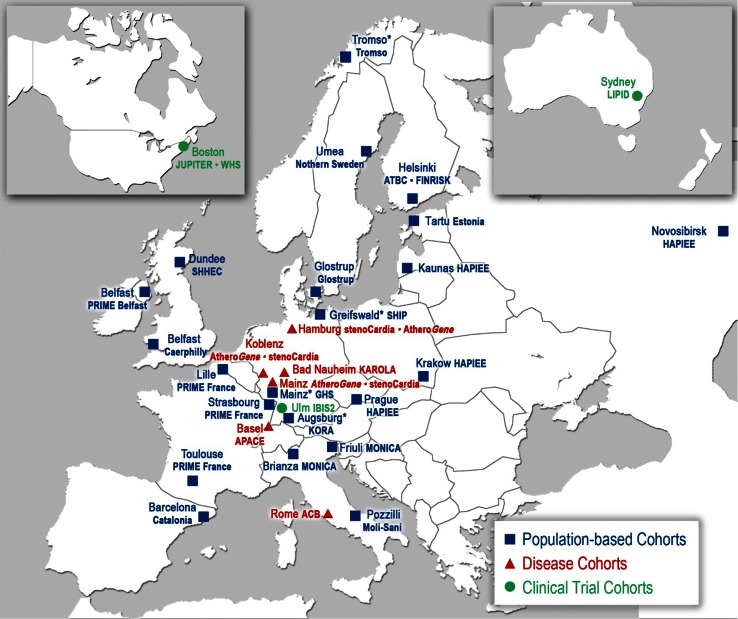
BiomarCaRE collaborating countries and cohorts across Europe. *Asterisk* indicates cohort with imaging data available

The collaborative BiomarCaRE consortium, an FP7 funded project, integrates the efforts of 25 academic institutions and five small/medium-sized, research intensive enterprises (SMEs) with a focus on cardiovascular biomarker research across Europe (Fig. 1). BiomarCaRE brings together large-scale epidemiological and clinical data and biomaterial resources across Europe and diverse expertise in epidemiology, clinical research, data and sample management, clinical chemistry, molecular biology, and biostatistics. The consortium capitalizes on in depth knowledge of omics-based candidate markers and integrates modern technologies of multiple biomarker assessment. It comprises 21 well-established prospective European population-based cohort studies, most of which were previously harmonized in the MORGAM Project [9], four cohorts of diseased subjects (disease cohorts, secondary prevention) and four clinical trials, totalling over 300,000 participants with a follow-up of over three million person years and storage of selected biomaterial of all participants in one central BiomaCaRE laboratory (University Heart Center Hamburg). This large individual-based database provides a unique opportunity to investigate the performance of established and novel biomarkers for Cardiovascular Risk Assessment across Europe.

An academic partner coordinates the consortium; the focus, however, is constructed around novel, emerging biomarkers and innovative technology and assays derived by SMEs. The consortium comprises an established research governance infrastructure, data harmonization and sharing arrangements, two central secure, access-managed phenotypic and biomarker data repositories as well as a core laboratory for biomarker measurements.

### Objective of BiomarCaRE

The objective of the BiomarCaRE consortium is to combine innovation in biomarker discovery with validation of newly identified and established biomarkers for CVD prediction across Europe. Ultimately, the BiomarCaRE consortium aims to develop a “European biomarker panel” for CVD prediction including classical risk factors and established and novel biomarkers. This will be achieved on the basis of SME-driven development of innovative technology and immunological/clinical chemistry assays and a large collaboration of well-defined European cohorts in primary and secondary prevention. The BiomarCaRE consortium measures success not only in terms of development of novel risk estimation models and their subsequent clinical utility, but also in terms of the development of novel, innovative technologies.

## Methods

### Design of BiomarCaRE

**Fig. 2:**
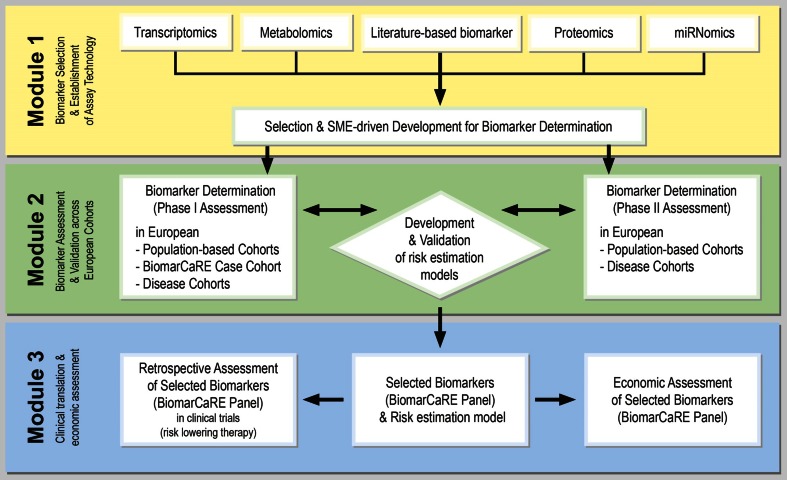
BiomarCaRE multi-modular concept. From biomarker selection and assay development (Module 1) to biomarker measurements and statistical analyses (Module 2) to clinical translation and economic assessment (Module 3). Module 1: Established and emerging biomarkers are prioritised according to their association with CVD risk, novel biomarkers are selected based on pre-existing non-publically available-omics datasets. Assay development is guided by SMEs and optimised for medium to high-throughput measurement. Module 2: The predictive value of biomarkers is assessed separately in population and disease based cohorts in a two phase approach; phase I assessment and phase II validation. Module 3 assesses the clinical utilisation of BiomarCaRE risk panels in clinical trials for their interaction with risk lowering therapy and develops a decision-analytical model to estimate long term cost-effectiveness of a primary or secondary preventive strategy guided by biomarker testing

The BiomarCaRE project is designed as a multi-modular study including: (1) biomarker selection based on omics discovery studies as well as literature, and (2) assay development (Module 1), (3) data harmonization of large-scale studies and (4) biomarker determination, analyses and validation (Module 2), and (5) biomarker assessment in clinical trials and (6) economic evaluation (Module 3). The main disease endpoints assessed in BiomarCaRE are incident acute coronary events, stroke, heart failure, atrial fibrillation, and diabetes mellitus. Other cardiovascular endpoints such as venous thrombosis will also be studied. Figure 2 gives a detailed overview about the multi-modular BiomarCaRE design.

### Cohorts, variables and biomaterial in BiomarCaRE

An overview of all cohorts, follow-up times and available biomaterial samples is outlined in Tables 1, 2, 3, 4, 5, 6 and a detailed description of the cohorts is provided in the Supplementary Material. For each cohort, ethical approval has been granted by the respective Ethical Committees and each participant gave informed consent. 

#### Population-based cohorts

The population cohorts originate from different countries of all European regions (Fig. 1). Each cohort is based on a well-defined population. Consistent measurement and data collection procedures were used for demographic, lifestyle, biological and clinical data. Some of the cohorts have ultrasound-based imaging and magnetic resonance imaging (MRI) data available (Table 1). Overall, the general population cohorts comprise 171,000 men and 129,000 women; 45,000 of them have serially sampled biomarkers available. The mean age at baseline is 51.2 (SD 12.7) years. All cohorts include subjects in mid-life, a time when early onset CVD manifests and where it is of importance to target improved risk estimation (Table 2). All cohorts followed-up the study participants prospectively for a range of 2.5–25 years for fatal and non-fatal acute coronary events and stroke and death. Some of the cohorts also followed-up heart failure, atrial fibrillation, type 2 diabetes, peripheral vascular disease, venous thromboembolism, and cancer.

**Table 1:** Population-based cohorts of BiomarCaRE

Study	Country	Cohort size	Mean follow-up time (years)	Incident events					Specific characteristics		Biomaterial		
				Acute coronary events	Stroke	Heart Failure	Atrial Fibrillation	Type 2 Diabetes	Imaging data	Nutrition data	Serum	EDTA Plasma	DNA
*PHASE 1*													
ATBC placebo	Finland	7,287	14	1,416	823	–	–	–	–	•*	•	–	•
FINRISK 97	Finland	8,444	14	412	303	594	479	624	–	•*	•	•	•
Glostrup 82–92	Denmark	7,582	23^¶^	828	747	673	649*	504	–	–	•	–	•
SHHEC	Scotland	16,000	21^¶^	1,882	869	1,132	1,118	874	–	•*	•	•	–
PRIME Belfast	United Kingdom	2,745	16	272*	102*	–	–	**	–	–	•	•	•
SHIP-TREND	Germany	4,308*	0	**	**	**	**	**	•	–	•	•	•
GHS	Germany	15,000	5	**	**	**	**	**	•	•*	•	•	•
KORA S3/S4	Germany	8,913	13^¶^	281	246	–	–	453	–	•*	•	•	•
PRIME France	France	7,855	10	291	90	–	–	–	–	•*	•	•	•
HAPIEE	Czech Republic	8,480	8	217*	209*	248*	–	–	–	•*	•	•	•
Brianza	Italy	4,932	21^¶^	222	133	–	–	–	–	–	•	–	•
Moli-Sani	Italy	24,325	4	163*	118*	833*	411*	350*	–	•	•	•	•
*PHASE 2*													
Tromsø	Norway	31,847	20^¶^	2,221*	1,367*	–	–	–	–	–	•	•	•
Northern Sweden	Sweden	10,517	23^¶^	1,268*	1,043*	1,251*	2,730*	800*	–	•*	•	•	•
ATBC treatment	Finland	21,846	14	4,327	2,382	–	–	–	–	•*	•	–	–
FINRISK 02/07	Finland	15,580	9^¶^	293	189	329	221	555	–	–	•	•	•
Glostrup 99/06	Denmark	10,984	11^¶^	221*	277*	–*	229*	359*	–	–	•	–	–
CAPS	United Kingdom	1,911	20^¶^	444*	268*	234*	–	–	–	•*	•	•	–
Estonian	Estonia	52,000	5	200*	200*	–	–	–	–	–	•	•	•
HAPIEE	Lithuania, Poland, Russia	26,522	6^¶^	578	339	–	–	–	–	•*	•	•	•
Friuli	Italy	1,786	4	12	8	–	–	–	–	–	•	–	•
Rome	Italy	4,489	10	74	81	–	–	–	–	–	•	•	•
Catalonia	Spain	5,505	10^¶^	76	81	–	–	–	–	•*	•	–	–

Population-based cohorts within phase 1 and phase 2 of the project are listed including information on cohort size, years of follow-up and number of incident cardiovascular events. Availability of imaging and nutrition data and types of biomaterial are given. Data are given with status in June 2014. * Indicates data not been harmonized to BiomarCaRE database yet; ** data not available yet; ^¶^shown is the longest average follow-up time (as there are several cohorts with different follow-up times); – indicates not available. ATBC, Alpha-Tocopherol, Beta-Carotene Cancer Prevention Study; FINRISK, FINRISK Study, Glostrup, Glostrup Study; SHHEC, Scottish Heart and Health Extended Cohorts; PRIME, Prospective Epidemiological Study of Myocardial Infarction Study; SHIP TREND, Study of Health in Pomerania; GHS, Gutenberg Health Study; KORA, Kooperative Gesundheitsforschung in der Region Augsburg; HAPIEE, Health, Alcohol and Psychosocial factors in Eastern Europe; Brianza, The Brianza Study; Moli-Sani, Moli-Sani Project; Tromsø, The Tromsø Study; Northern Sweden, The Northern Sweden MONICA Study; CAPS, Caerphilly Prospective Study; Estonian, Estonian Genome Center of the University of Tartu (EGCUT)—The Estonian Biobank; Friuli, MONICA Friuli; Rome, The Rome Study (Malattie Aterosclerotiche Istituto Superiore di Sanità (MATISS); Catalonia, The MONICA-Catalonia Study

**Table 2:** Overall characteristics of the BiomarCaRE population cohorts

*Characteristics*	
Number of cohort studies^a^	21
Number of individuals	300,000
Years of baseline examinations	1982–2012
Men (%)	57 %
Mean age (SD) at baseline (years)	51.2 (12.7)
Smoker (%)	38 %
Diabetes (%)	5 %
Hypertension (%)^b^	42 %
*Number of incident endpoints during follow*-*up*	
Acute coronary events^c^	13,700
Stroke^d^	8,400
Heart failure^e^	3,800
Atrial fibrillation^f^	2,900
Type 2 diabetes^g^	3,400
Overall Death^h^	35,600

Number are given for distinct individuals, excluding repeated measurements

^a^Status as of June 2014

^b^Blood pressure >140/90 or under treatment

^c^Definite or possible myocardial infarction or coronary death, or unstable angina pectoris. The number is available for 33 cohorts, estimated for 2 cohorts. Excluding individuals with history of cardiovascular disease

^d^The number is available for 34 cohorts, estimated for 1 cohort. Excluding individuals with history of cardiovascular disease

^e^The number is available for 11 cohorts, estimated for 1 cohort. Excluding individuals with history of heart failure

^f^The number is available for 10 cohorts. Excluding individuals with history of atrial fibrillation

^g^The number is available for 13 cohorts, estimated for 1 cohort. Excluding individuals with history of type 1 or type 2 diabetes

^h^The number is available for 37 cohorts, given for 1 cohort

The harmonised baseline variables include measured systolic and diastolic blood pressure, blood lipids, weight, height, waist and hip circumference, and questions on smoking, disease history and medication for hypertension, dyslipidaemia and diabetes. Assessment and validation of the end-points varied between cohorts (Kulathinal et al. [20] ). Acute coronary events are harmonized to categories: definite or possible myocardial infarction or coronary death, unstable angina pectoris, cardiac revascularization, or unclassifiable death, where the data are insufficient for the other coronary diagnoses and there is no evidence of other causes. The categories can be combined to define different end-points for analysis. Stroke has been characterized as ischaemic stroke, intracerebral haemorrhage, and subarachnoid haemorrhage, although reliable diagnostic information for the subtyping is not always available, in particular for the early years of follow-up. Follow-up for heart failure, atrial fibrillation, type 2 diabetes, peripheral vascular disease, venous thromboembolism and cancer were usually based on linkage with national hospitalization registers, other administrative registers and cancer registers.

#### Disease cohorts

In patients with manifest CVD, in particular after an acute coronary syndrome (ACS), a number of clinical variables have been proven useful for risk assessment. These variables have been integrated into various scores like the Thrombolysis in Myocardial Infarction (TIMI) Risk Score, and the Global Registry of Acute Coronary Event (GRACE) Score. Based on such parameters fairly reliable prediction can be made not only short-term but even over a period of more than 5 years [11]. However, despite such routinely available variables, further improvement of risk assessment in this high-risk group is of paramount importance because available treatment options should be tailored to the individual patient’s risk. The BiomarCaRE consortium offers the opportunity to directly translate population-derived biomarker information into the clinical setting of manifest CVD and ACS. Within the consortium four disease cohorts comprising almost 9,000 individuals with either angiographically proven stable coronary artery disease (CAD) mostly after myocardial infarction or individuals presenting with suspected acute ACS have been harmonized for CVD history, clinical symptoms and classical cardiovascular risk factor information. The clinical endpoints followed up in the disease cohorts are (1) total mortality, (2) cardiac death, (3) cardiovascular death including fatal cardiovascular events such as cerebrovascular diseases, and (4) cardiovascular events including the combined endpoint of cardiovascular death and non-fatal myocardial infarction (Table 3). The mean age is 61.5 (SD 12.4) years. Follow-up varies between 6 months and 7.5–10 years. Baseline variables are outlined in Table 4. Combining data from different diseases cohorts in BiomarCaRE, in particular from the AtheroGene and KAROLA studies with long-term follow-up, guarantees sufficient power to reliably describe biomarker associations with recurrent cardiovascular events and to test the applicability of a marker panel in diverse clinical settings.

**Table 3:** Disease cohorts of BiomarCaRE

Study	Country	Size	Follow-up time (years)	Endpoints				Biomaterial		
				Total mortality	Cardiac death	Cardiovascular death	Cardiovascular events	Serum	EDTA Plasma	DNA
AtheroGene	Germany	3,476	7.5	386	244	260	460	•	•	•
KAROLA	Germany	1,204	10	184	91	103	162	•	•	•
APACE	Switzerland	2,248	2.2	174	71	73	534	•	•	–
stenoCardia	Germany	1,818	0.5	34	0	7	38	•	•	•
Rome ACB*	Italy	500^a^	–	–	–	–		•	•	–

The disease cohorts of the project are listed including information on cohort size, years of follow-up and availability of biomaterial. Endpoint cardiovascular death includes fatal cardiovascular events such as cerebrovascular diseases; Endpoint cardiovascular events is a combined endpoint of cardiovascular death and non-fatal myocardial infarction * Median follow-up time. KAROLA, Langzeiterfolge der Kardiologischen Anschlussheilbehandlung; APACE, Advantageous Predictors of Acute Coronary Syndromes Evaluation Study; stenoCardia, Study for evaluation of newly onset chest pain and rapid diagnosis of myocardial necrosis

^a^Data not been harmonized to BiomarCaRE database yet

**Table 4:** Overall characteristics of the BiomarCaRE disease cohorts

*Characteristics*	
Number of cohorts	4
Number of individuals	8,746
Years of baseline examinations	1996–2012
Men (%)	73
Mean age (SD) at baseline (years)	61.5 (12.4)
Smoker (%)	23
Type 2 diabetes (%)	19
Hypertension (%)	69

The numbers represent individuals at baseline, excluding repeated measurements. Disease cohort Rome ACB not included as data not been harmonized to BiomarCaRE database yet

#### Clinical trials

Various biomarkers may be affected by contemporary pharmacological strategies as seen for example in the case of hsCRP and statin treatment [24]. This biomarker may be lowered by 40 % when highly potent statins are given. Thus, it may be important to test the effects of various therapies on our finally selected biomarkers. Based on access to biobanks of several large clinical trials, BiomarCaRE will test biomarkers as measures of therapeutic response related to aspirin in women (Women´s Health Study), LDL-Cholesterol lowering by statins (The Long-Term Intervention with Pravastatin in Ischaemic Disease (LIPID) Study and The Justification for the Use of Statins in Primary Prevention: An Intervention Trial Evaluating Rosuvastatin (JUPITER) trial), and anti-inflammatory treatment with an Lp-PLA2 inhibitor (darapladib) [Integrated Biomarker and Imaging Study-2 (IBIS-2)] (Table 5).

**Table 5:** Clinical trials of BiomarCaRE

Study	Setting	Size	Intervention	Median follow up time (years)	Primary endpoint
LIPID	Secondary prevention	9,014	Pravastatin (40 mg)	6.1	CHD Death Non-fatal MI
JUPITER	Primary prevention	17,802	Rosuvastatin (20 mg)	1.9	MI Stroke Cardiovascular death Hospitalization for UAP
WHS	Primary prevention (women only)	39,876	Aspirin (100 mg) every other day	10.1	Non-fatal MI Non-fatal stroke Cardiovascular death
IBIS-2	Secondary prevention	330	Darapladib (160 mg)	1	Coronary atheroma Progression by IVUS Plaque deformability by Palpography hsCRP

Clinical trials of the project are listed including information on trial setting, size, intervention, duration and primary endpoint of trial. LIPID, The Long-Term Intervention with Pravastatin in Ischaemic Disease Study; JUPITER, The Justification for the Use of Statins in Primary Prevention: An Intervention Trial Evaluating Rosuvastatin Trial; WHS, The Women’s Health Study; IBIS-2, Integrated Biomarker and Imaging Study-2; hsCRP, high-sensitivity measured C-reactive protein

### Biomaterial

A prerequisite for all BiomarCaRE cohorts is the availability of biomaterial samples, such as plasma, serum and genomic DNA. Furthermore, innovative biomaterial such as plaque tissue is also available in some of the BiomarCaRE cohorts. The Cohort Centres select samples from their repositories and transfer them to the BiomarCaRE central laboratory in Hamburg, Germany, where the measurement of all established and novel biomarkers is performed.

#### Selected established, emerging, and “omics-based” biomarkers, and assay development by SMEs

**Fig. 3:**
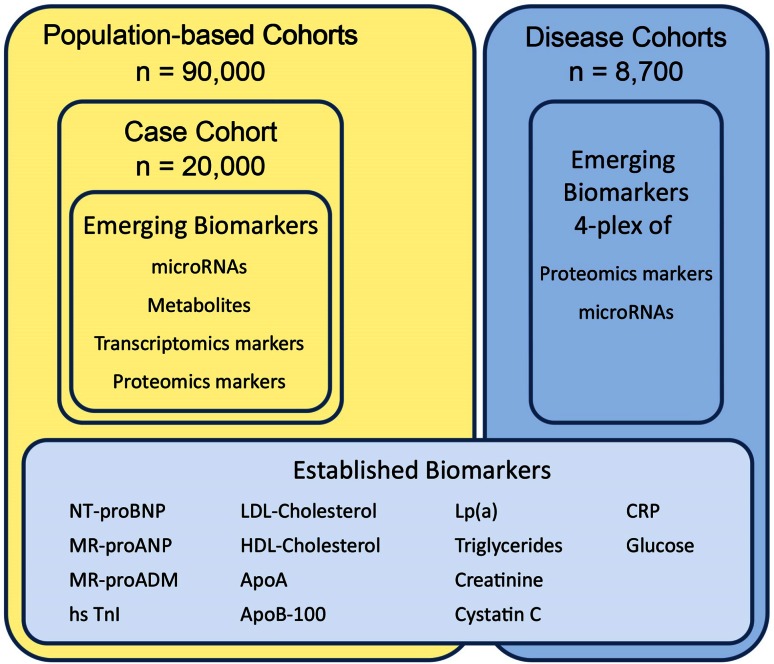
Emerging and established biomarkers measured in phase 1 of the population-based cohorts (including a case-cohort set) and the disease cohorts within BiomarCaRE. NTproBNP, N-terminal pro B-type natriuretic peptide, MR-proANP mid-regional pro atrial natriuretic papetide; MR-proADM, mid-regional pro adrenomedullin; hsTnI, high-sensitivity assayed Troponin I; ApoA, apolipoprotein A1; ApoB100, apolipoprotein B100; Lp(a) Lipoprotein a; CRP, C-reactive protein

In Module 1 of the project (Fig. 2), established biomarkers are selected according to their association with the risk of CVD, and based on the expertise and knowledge of the BiomarCaRE consortium and the public domain. The BiomarCaRE consortium has already determined an extensive panel of biomarkers within the MORGAM Biomarker substudy [4]. Key established biomarkers to be addressed are high-sensitivity troponins (hsTn), natriuretic peptides, high-sensitivity C-reactive protein (CRP), lipids, and further markers of cardiomyocyte micronecrosis, inflammation, and renal function (Fig. 3). The concept of biomarkers reflecting different underlying pathways to disease progression combined in multiple biomarker risk scores has already been applied in several population-based studies [4, 14, 21, 25, 39] as well as in disease cohorts [26]. Several of those markers, in particular hsTn, hsCRP as well as growth differentiation factor 15 (GDF-15) have been proposed to guide therapy [1, 17, 24]. Commercially available assays are used to determine the levels of established biomarkers in the biomaterial of study participants.

Selection of novel biomarkers is based on pre-existing but not publicly available –omics datasets on quantitative proteomics, transcriptomics, metabolomics, and miRNomics. The emerging value of circulating microRNAs (miRNAs) and circulating cells such as microparticles as specific and sensitive biomarkers has been discussed by several groups [10, 16, 33, 34]. In addition, assessment of the transcriptome can identify disease-related mRNA signatures which might be translated into clinically useful biomarkers [22, 31, 37]. Apart from proteomics and transcriptomics approaches, metabolite profiling in large, well-phenotyped cohorts is very rapidly emerging in the cardiovascular field [23, 27, 28, 35] and provides access to a biomolecular repertoire not covered by other omics applications.

The novel biomarkers are disclosed by the academic and SME partners. The SMEs introduce the technology and guide the development of the innovative assays needed for the measurement of novel biomarkers. In addition, the SMEs optimise the required technology so that medium- to high-throughput measurements become possible. Assay validation has been carried out before incorporation by the BiomarCaRE laboratory. The following technologies are established to apply omics-based discovery results in population based and disease cohorts: a) A microparticle-based protein biomarker panel will be validated in the disease cohorts to confirm their value as markers in the prediction of secondary manifestations of CVD [16]. For this purpose, development of a 4-plex assay based on a Luminex platform is ongoing An Enzyme-Linked Immunosorbent Assay (ELISA) is being developed within BiomarCaRE to assess cardiovascular risk, specifically in women. To investigate circulating miRNAs, a highly versatile multiplex detection platform to support the multiplex measurement of circulating miRNAs is being developed. Candidate genes from transcriptomics datasets of BiomarCaRE partners [38] that may be related to cardiovascular events or phenotypes have been selected as biomarker targets, and ELISAs for higher throughput measurements are being developed. The Absolute*IDQ* p180 kit (www.biocartes.com), which identifies and quantifies 185 metabolites by mass spectrometry, will be used to profile the metabolome. The metabolome profile and the transcriptomics-based biomarkers including miRNA will be assessed within BiomarCaRE in the case cohort setting (n = 20,000). Figure 3 provides an overview of established and emerging biomarkers measured in BiomarCaRE.

In addition to the emerging, omics-based biomarkers, novel biomarkers from the literature, such as biomarkers for heart failure (e.g. soluble isoforms of the IL-1 receptor family member (sST2) and Galectin-3) are being selected. An overview of all established and emerging, literature-based biomarkers to be measured in BiomarCaRE is given in the Supplementary Material.

### Measurement of biomarkers

In Module 2 of the project (Fig. 2), a two-phase approach for the biomarker measurement is used: in phase 1, established and novel biomarkers are determined in a set of population cohorts from different European regions, including 90,000 subjects with 6,000 incident acute coronary events, 3,800 incident strokes, 3,500 incident cases of heart failure, 2,600 cases with incident atrial fibrillation and 2,800 incident type 2 diabetes diagnoses during follow-up (Table 1). In addition, markers which are considered as most innovative but “high risk” markers, such as metabolites, miRNA and other novel markers derived from omics approaches will be measured in a case-cohort set of up to 20,000 population-based subjects including 4,500 incident acute coronary events. This case-cohort design reduces the number of biomarker measurements without substantially reducing statistical power [19]. The case-cohort study involves a random subsample of the selected population cohorts and in addition all incident cases of these cohorts (Table 6). Independent of the population-based cohorts, the selected biomarkers are also measured in the four diseased cohorts (n = 8,746, Table 3).

**Table 6:** Number of cases and non-cases in the individual case cohort sets of the cohorts selected for the BiomarCaRE case-cohort study

Study	Country	Number of incident cases					Number of non-cases
		Coronary heart disease	Stroke	Heart failure	Atrial fibrillation	Type 2 diabetes	
Glostrup	Denmark	821	744	608	603	526	2,209
ATBC Placebo	Finland	1,414	822	–	–	–	2,068
FINRISK 97	Finland	354	260	442	307	525	1,384
PRIME France	France	286	90	–	–	–	414
KORA S3/S4	Germany	252	224	–	–	390	910
Brianza	Italy	218	119	–	–	–	369
PRIME Belfast	United Kingdom	185	53	–	–	–	282
SHHEC	Scotland	940	411	489	475	477	2,055
Total	4,470	2,723	1,539	1,385	1,918	9,691	

Numbers are given as status in June 2014. ATBC, Alpha-Tocopherol, Beta-Carotene Cancer Prevention Study; FINRISK, FINRISK Study, Glostrup, Glostrup Study; SHHEC, Scottish Heart and Health Extended Cohorts; PRIME, Prospective Epidemiological Study of Myocardial Infarction Study; KORA, Kooperative Gesundheitsforschung in der Region Augsburg; Brianza, The Brianza Study

In phase 2, the most promising biomarkers from phase 1 are further determined in 130,000 subjects from European population cohorts (Table 1). Biomarkers measured within the BiomarCaRE project are depicted in Fig. 3.

### Statistical concepts

**Fig. 4:**
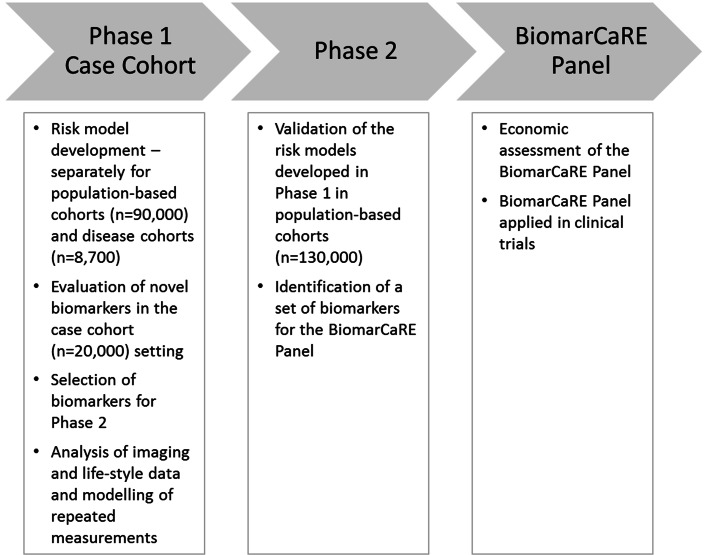
Overview of data analyses performed in phases 1 and 2 of BiomarCaRE

BiomarCaRE comprises two closely interacting data centres: one centre that is responsible for the analyses of all population-derived biomarkers which is based at the National Institute for Health and Welfare (THL) in Helsinki; Finland. This data centre has a long-standing and unparalleled experience of epidemiological data harmonization and central data management and analyses of the MORGAM Project [9] and its predecessor, the WHO MONICA Project (Tunstall-Pedoe and Project [32]. The second data centre is based at the University at Lübeck, Germany and is responsible for the analyses of biomarkers derived from the diseased cohorts. Data obtained in clinical trial repositories will be analysed directly at the respective partner site. Figure 4 illustrates and summarizes analyses performed in phases 1 and 2.

As biomarkers may have different effects in healthy and diseased subjects, the population-based and diseased cohorts will be analysed separately. The statistical analysis for both types of cohorts will begin with univariate testing of each biomarker with adjustment for classical cardiovascular risk factors. Significant biomarkers are selected for subsequent analysis in phase 2 cohorts for validation.

The risk models are developed using Cox regression analysis, taking the case-cohort sampling into account [19] for some biomarkers. The modelling steps include initial checking of model assumptions and the stability and parsimony of biomarker selection. As a complementary approach for biomarker selection, we will apply random survival forests [13, 15] which is a machine learning technique to determine important variables for the prediction of individual survival times. Performance of the risk estimation models will be cross-validated using calibration, discrimination indices and graphs and net reclassification improvement. The developed predictive models are compared with established risk score models, such as the SCORE [6].

The impact of the biomarkers and the predictive models developed in phase 1 is externally validated using the prospective population-based cohorts and biomarker data from phase 2. These analyses will result in the construction of a risk model including classical risk factors and a combination of the validated biomarkers (BiomarCaRE Panel). As a secondary subsidiary aspect, the cross-sectional association between biomarkers and cardiac imaging data and between biomarkers and health behaviour data will be assessed in individual studies where data are available. Furthermore, joint modelling of repeated measurements of the biomarkers and survival data will be performed for selected cohorts.

To analyze the effects of various pharmacological interventions on the BiomarCaRE panel of selected biomarkers stepwise statistical strategies will be applied in the clinical trials.

In a first step, the predictive value of the BiomarCaRE panel will be assessed in samples from the clinical trials, adjusted for medication and stratified by intervention group. In a second step, the change in biomarker levels from baseline to (on average) 1 year on treatment will be assessed. Finally, the treatment effect will be analyzed according to biomarker levels at baseline (high vs. low).

#### Proposed imputation strategy for the BiomarCaRE project

Clinical and biomarker variables are often incomplete due to e.g. sample unavailability or assay failure. Data analysis using only complete sets for multiple variables would lead to loss of available information, thus to reduced statistical power. Furthermore, the results of such analysis can be biased. One approach to make the best use of available data is to predict the missing information from the observed data. This process minimizes potential bias, and is termed multiple imputation [8] and will be used in BiomarCaRE analysis when appropriate. A common strategy in clinical epidemiology is to exclude variables with more than 20 % missing data because of the high potential for a severe bias. If less than 5 % of the data are missing, substantial bias is unlikely [2]. If 20 % of the biomarker measurements are missing, the biomarker may be excluded from a multimarker analysis, depending on what is known about the reason for the missing data. Decisions will be made on a case by case basis.

### Economic assessment of single and combined biomarker application

In the third module of the project (Fig. 2), the focus will be on the clinical utilisation and cost-effectiveness of the selected biomarkers, and their single and joint application. Selected biomarkers from phase 2 will be measured in samples of clinical trials to test their interaction with risk lowering therapy (Table 5) and a decision-analytic model will be developed to estimate long-term effectiveness and cost-effectiveness of a primary or secondary preventive strategy based on additional risk assessment using these biomarkers.

Decision-analytic modelling allows for the combination of empirical evidence on improvements in risk prediction and published evidence on short- and long-term effects of health are interventions to estimate the health effects on patient-relevant endpoints such as life expectancy or quality-adjusted life expectancy. Total costs of a strategy will then be estimated by combining cost savings through prevention of advanced disease and costs for the biomarker panel tests, diagnostic tests and work-up, drug treatment and monitoring of patients. A decision-analytic state-transition (Markov) model will be used, as this model type suits decision problems that can be described in specific health states and transition probabilities [5]. The analysis will be performed as a cohort simulation or as a microsimulation depending on the number of covariates to be tracked in the simulation [30], and deterministic and probabilistic sensitivity analyses will be performed. The long-term effectiveness of the biomarker-guided strategies will be expressed in life years and quality-adjusted life years (QALY). Cost-effectiveness will be expressed as incremental cost-effectiveness ratios, that is, the additional costs divided by gains in effectiveness [29]. In particular, we will systematically vary biomarkers and cut-offs in our model to identify candidate strategies for effective and cost-effective biomarker testing that can be investigated in future clinical trials.

## Discussion

The BiomarCaRE project aims to determine the additional value of multiple (new) biomarkers to improve risk estimation of CVD related events in Europe. The project is unique in terms of its dimension, targeting of novel biomarkers based on -omics technology, and the evaluation of the impact of a multiple biomarker score in large prospective population cohorts across different European regions. The project integrates modern, mainly SME driven molecular technologies with epidemiological approaches supplemented by economical assessment. Evaluation of biomarkers on a large-scale using high sensitivity tests improves the precision of the risk estimates. A major strength of the project is the derivation of novel risk estimation models and assessment in a variety of settings ranging from general populations to those with disease and hypothesis-generating clinical trials. The resulting models may prove most useful in refining risk in subgroups of the population that could benefit from early treatment.

To date, it is not clear to what extent single or multiple biomarkers improve CVD prediction and how they provide additional information for therapy selection and guidance. Ideally, selection of biomarkers and generation of multi-marker panels would be based both on biological plausibility (and independent causal effects) and empirical evidence on their influence on disease risk. However, the causal relevance of many predictive biomarkers of CVD has yet to be fully established, but this may not necessarily be required for risk prediction. Recent research has demonstrated that scores comprising biomarkers that show low to moderate correlation between each other and represent different biological pathways could improve discrimination and calibration [4, 14, 25]. As the number of pathways known to contribute to cardiovascular risk is expanding, selection of biomarkers from different pathways is challenging. The added value contributed by the BiomarCaRE consortium is the ability to capitalise on proteomics, metabolomics, transcriptomics and miRNAomics approaches which may yield promising candidate markers from novel pathways which can be compared robustly to other established and emerging biomarkers. This is based on newly developed technologies to apply innovative biomarkers on a large-scale basis. Furthermore, the project will extend our knowledge of biomarker-driven prognostication to encompass the wider cost-effectiveness implications using a simulation approach.

The ultimate goal of BiomarCaRE, building a robust European risk score, would greatly facilitate individualized risk estimation, screening, and diagnosis of CVD. The novel risk score would carry the potential to change existing guidelines and could thus have significant public health impact.

Current research further suggests that considerable differences exist between women and men with respect to disease mechanisms and outcomes. However, small number of women developing manifest disease in population studies has often made it difficult to assess sex differences. Furthermore, women are not adequately represented in clinical trials. Thus, gender-specific analyses have been mostly underpowered. Because of the increasing incidence of CVD in women, there is a need to gain insight into gender-specific biomarker properties. Within BiomarCaRE the large cohort size will allow gender-based subgroup analyses with sufficient power.

### Expected outcome and perspective of personalized medicine in Europe

The BiomarCaRE project will be in a unique position to carefully design pragmatic randomized controlled trials to understand the clinical implications of reclassification, in particular to establish the safety and effectiveness of deferring treatment for those reclassified into a lower risk category based on biomarkers. Further, the study will give clues to whether any intensified intervention in those reclassified to higher risk categories might ultimately result in improved clinical outcomes. In this regard, BiomarCaRE has already initiated the planning of the “post” BiomarCaRE phase that will prospectively validate clinical utilisation in personalised medicine, by proposing the execution of a biomarker guided clinical trial utilizing the results of the BiomarCaRE consortium.

### Electronic supplementary material

Below is the link to the electronic supplementary material.

Supplementary material 1 (DOCX 127 kb)

## Members of the BiomarCaRE study group

Medical University Hamburg Eppendorf, Hamburg, Germany: Stefan Blankenberg, Tanja Zeller, Simone Schnella, Renate B Schnabel, Francisco Ojeda, Sebastian Appelbaum, Johannes Neumann; Mahir Karakas, Christian Schulte, Annika Jagodzinski

National Institute for Health and Welfare, Helsinki, Finland: Kari Kuulasmaa, Veikko Salomaa, Jarmo Virtamo, Satu Männistö, Matti Niemelä, Tarja Tuovinen Zygimantas Cepaitis, Ari Haukijärvi, Aki Havulinna, Olli Saarela, Jukka Kontto, Arto Pietilä

Cavadis B.V., Utrecht, The Netherlands: Heico Breek, Herfita Agustiandari

Universitair Medisch Centrum, Utrecht, The Netherlands: Gerard Pasterkamp, Hester den Ruijter, Marten Siemelink

Helmholtz Zentrum Muenchen, Germany: Barbara Thorand, Annette Peters

Biocrates Life Sciences AG, Innsbruck, Austria: Cornelia Röhring; Manuel Kratzke

University Lübeck, DE: Andreas Ziegler, Arne Schillert, Andrea Senft

University for Health Sciences, Medical Informatics and Technology, Hall i.T., Austria: Annette Conrads-Frank, Uwe Siebert, Petra Schnell-Inderst, Felicitas Kühne

Medizinische Hochschule Hannover, Germany: Kai C. Wollert, Tibor Kempf

Fleet Bioprocessing Ltd., Hartley Wintney, UK: Alastair Dent, Julian Duncan, Nic Christofides, Lindsay Robertson

Biocartis SA, Lausanne, Switzerland: Eva Servoli, Patrick van den Bogaard

Research Network Services Ltd., Berlin, Germany: Eric Werner

Universitätsmedizin der Johannes Gutenberg-Universität Mainz, Germany: Philipp Wild, Karl Lackner, Thomas Münzel

University of Tromsø, Norway: Ellisiv B Mathiesen, Inger Njølstad

Università Cattolica del Sacro Cuore, Roma, Italy: Luigi Marzio Biasucci

IRCCS Istituto Neurologico Mediterraneo Neuromed, Pozzilli, Italy: Licia Iacoviello, Simona Costanzo, Augusto Di Castelnuovo, Giovanni de Gaetano

Ernst-Moritz-Arndt Universität Greifswald, Germany: Henry Völzke, Roberto Lorbeer

University College London, UK: Martin Bobak

Institute Pasteur de Lille, France: Jean Dallongeville, Jean Ferrières, Dominique Arveiler, Philippe Amouyel

Catalan Institute of Cardiovascular Sciences, Barcelona, Spain: Susana Sans

The Queen’s University of Belfast, UK: Frank Kee, Maria Hughes, Felicity Lamrock, Karen Cairns

Umeå Universitet, Sweden:Stefan Söderberg

Research Centre for Prevention and Health, Glostrup, Denmark: Torben Jørgensen, Allan Linneberg, Anders Borglykke

University of Insubria, Varese, Italy: Marco Ferrario, Giovanni Veronesi

University of Dundee, UK: Hugh Tunstall-Pedoe, Jill Belch, Gwen Kennedy

Universitätsspital Basel, Switzerland: Christian Müller, Raphael Twerenbold, Beate Hartmann

Universitätsklinikum Ulm, Germany: Wolfgang Koenig, Dietrich Rothenbacher

Hamilton Health Sciences Corporation, Canada: Sonia Anand, Salim Yusuf

University of Sydney, Sydney, Australia: John Simes, Andrew Tonkin

Harvard Medical School, Brigham and Women’s Hospital, Boston, USA: Brendan Everett, Paul Ridker
