# Supplementary material for: BiomarCaRE: rationale and design of the European BiomarCaRE project including 300,000 participants from 13 European countries
Source: Eur J Epidemiol. 2014 Sep 20;29(10):777–90. doi: 10.1007/s10654-014-9952-x (PMC4197377; doi:10.1007/s10654-014-9952-x)
Supplement: Supplementary file 1 — Supplementary material 1 (DOCX 127 kb) [file 10654_2014_9952_MOESM1_ESM.docx]

**Supplementary Material**

**BiomarCaRE - Rationale and Design of the European BiomarCaRE Project including 300 000 participants from 13 European countries**

**Short Titel: The BiomarCaRE Study**

**Journal: European Journal of Epidemiology**

Tanja Zeller^1,2^*, Maria Hughes^2,3^*, Tarja Tuovinen^4^, Arne Schillert^2,5^, Annette Conrads-Frank^6^, Hester den Ruijter^7^, Renate B Schnabel^1,2^, Frank Kee^3^, Veikko Salomaa^4^, Uwe Siebert^6,8^, Barbara Thorand^9,10^, Andreas Ziegler^2,5,11^, Heico Breek^12^, Gerard Pasterkamp^7^, Kari Kuulasmaa^4^, Wolfgang Koenig^13^, Stefan Blankenberg^1,2^, on behalf of the BiomarCaRE consortium

^1^ University Heart Centre Hamburg, Clinic for General and Interventional Cardiology, Hamburg, Germany

^2^ German Centre for Cardiovascular Research (DZHK) Partner Site Hamburg/Lübeck/Kiel

^3^ UK Clinical Research Collaboration Centre of Excellence for Public Health, Queens University of Belfast, Northern Ireland

^4^ National Institute for Health and Welfare, Helsinki, Finland

^5^ Institut für Medizinische Biometrie und Statistik, Universität zu Lübeck, Universitätsklinikum Schleswig-Holstein, Campus Lübeck, Lübeck, Germany

^6^ Department of Public Health and Health Technology Assessment, UMIT - University for Health Sciences, Medical Informatics and Technology, Hall i.T., Austria

^7^ Universitair Medisch Centrum, Utrecht, The Netherlands

^8^ Harvard School of Public Health and Harvard Medical School, Boston, MA, USA

^9^ Helmholtz Zentrum München, German Research Center for Environmental Health, Institute of Epidemiology II, Neuherberg, Germany

^10^ German Centers for Cardiovascular Research (DZHK) Partner Site Munich

^11^ Zentrum für Klinische Studien Lübeck, Universität zu Lübeck, Germany

^12^ Cavadis B.V., Utrecht, The Netherlands

^13^ University of Ulm Medical Centre, Department of Internal Medicine II-Cardiology, Germany

* Both authors contribute equally

**Corresponding authors**:

Stefan Blankenberg; Tanja Zeller

Email: s.blankenberg@uke.de

Email: t.zeller@uke.de

Phone: +49 40 7410 56575

Fax: +49 40 7410 40194

1. **Overview and Description of BiomarCaRE Cohorts**
2. **Funding and Acknowledgements of Cohorts**
3. **Description of established and emerging, literature-based biomarkers**

**Overview and Description of BiomarCaRE Cohorts**

All study participants provided written, informed consent

**General Population Based Cohorts**

**Alpha-Tocopherol, Beta-Carotene Cancer Prevention (ATBC) Study**: The ATBC Study is a cohort of male smokers (aged 50-69 years) recruited in 1985-1988. The participants were screened from the total male population aged 50-69 years living in southern and western Finland (N = 290,406) through a postal questionnaire on their smoking habits and willingness to participate. Smokers of at least 5 cigarettes per day and willing to participate were invited to the local study center for further evaluation of their eligibility. Men were excluded due to a history of cancer or serious disease limiting their ability to participate or use of excess vitamin supplements. In all, 29,133 men were randomized to receive in a controlled trial design either alpha-tocopherol, or beta-carotene, or both, or placebo, and of them 99.9% donated a baseline serum sample (Round 1).  In 1986-1993 a random intermediate serum sample was collected from 3701 participants (12.7%) known as Round 2. A whole blood sample was collected in 1992-1993 from 70.2% of the original cohort comprising 20,167 participants (Round 3). Men were prospectively followed up for cardiovascular and cancer endpoints until 2004 using record linkage to national Hospital Discharge Register, Cancer Registry, and Register of Causes of Death with validation described in (1, 2, 3). <http://www.thl.fi/publications/morgam/cohorts/full/finland/fin-atba.htm> <http://atbcstudy.cancer.gov/>

**Caerphilly Prospective Study (CAPS):** The Caerphilly cohort is a prospective population based cohort from Wales United Kingdom (4). Men aged 56-70 years in 1989-93 were selected from the general population of Caerphilly in South Wales (population 40,000) based on their date of birth from electorial registers and private census. Phase 3 (N=2,171) was collected in 1989-93 and re-examined 10 years later in 2002-2004 (Phase 5, N=1,225) taking repeated measurements of several risk factors and biomarkers using blood samples stored at -70°C. Follow-up for deaths was performed by the Office of National Statistics and for non-fatal cardiovascular events using data linkage of hospital and GP records with validation by a study medical committee. Follow-up has been completed to February 2012. <http://www.thl.fi/publications/morgam/cohorts/full/uk/unk-caea.htm>

Estonian Genome Center of the University of Tartu (EGCUT) – The Estonian Biobank: The Estonian Biobank is a population-based biobank of the Estonian Genome Center of the University of Tartu (EGCUT). The project is conducted in accordance with the Estonian Gene Research Act ([www.biobank.ee](http://www.biobank.ee)) and all subjects have been recruited randomly, on voluntary basis by general practitioners and physicians in hospitals. As of June 2013 the number of individuals is 51 713, which represents about 5% of Estonia’s adult population. Phenotyping of subjects (18-103 years of age) was performed by Computer Assisted Personal interview (CAPI), including personal and genealogical data, educational and occupational history and lifestyle factors. Follow up of incident fatal and non-fatal coronary heart disease and stroke events of a subset of the cohort is on-going as our database is being linked with the national healthcare registries and regional and central hospital databases. Events are recorded according to the International Classification of Diseases (ICD-10). All subjects provided written informed consent prior to participation and the approval for the study was granted by the Ethics Review Committee on Human Research at the University of Tartu (5). [www.biobank.ee](http://www.biobank.ee)

**FINRISK:** The FINRISK study is a series of population-based cardiovascular risk factor surveys carried out every five years in five (or six in 2002) districts of Finland, including North Karelia, Northern Savo (former Kuopio), Southwestern Finland, Oulu Province, Lapland province (in 2002 only) and the region of Helsinki and Vantaa. A stratified random sample was drawn for each survey from the national population register, the age-range was 25-74 years. All individuals enrolled in the study received a physical examination, a self-administered questionnaire, and a blood sample was drawn. In 1997, altogether 11,500 individuals were invited and 8,444 (73%) participated in the clinical examination. Approximately 8,000 individuals participated in 2002 and 6,000 in 2007. During follow-up the National Hospital Discharge Register, the National Causes of Death Register and the National Drug Reimbursement Register were used to identify endpoints. At the moment, the follow-up extends until Dec. 31^st^, 2010, i.e., 14 years for the FINRISK 1997 cohort. The Coordinating Ethics Committee of the Helsinki and Uusimaa Hospital District approved the study, which followed the declaration of Helsinki. All subjects gave written informed consent. <http://www.thl.fi/publications/morgam/cohorts/full/finland/fin-fina.htm>

**Gutenberg Health Study (GHS):** Gutenberg Health Study (GHS): The Gutenberg Health Study (GHS) is designed as a community-based, prospective, observational, single-center cohort study in the Rhine-Main area of Western Germany (6). The sample was drawn randomly from the governmental local registry offices in the city of Mainz and the district of Mainz-Bingen. The sample was stratified 1:1 for sex and residence (urban and rural) and in equal strata for decades of age. Individuals between 35 and 74 years of age were enrolled. Exclusion criteria were insufficient knowledge of the German language and physical or psychological inability to participate in the examinations at the study centre. Baseline examination of 15,000 study participants was performed between 2007 and 2012. A 2.5 year follow-up conducted as a telephone interview started in 2009. Since 2012, the 5-year follow up has been achieved through record linkage, including a second visit at the study centre with extensive medical examination and re-sampling of the biomaterial which is ongoing. <http://www.gutenberghealthstudy.org/>

**Glostrup Study:** The Glostrup cohorts are five prospective population based cohorts from 11 municipalities from urban Glostrup of Copenhagen, Denmark. Random sampling based on the national population register, stratified by sex and year of birth was used for cohorts 1, 2 and 3. Cohort 1 consists of men and women aged 30-60 years having two repeated measurements of risk factors and biomarkers. Round 1 of cohort 1 was collected in 1982-1984 (N=3,785) and re-examined in 1987-1988 (Round 2, N=3,000) and 1993-1994 (Round 3, N=2656) (7). Cohort 2 (N=1,504) was examined in 1986-1987 and cohort 3 (N=1,624) was examined in 1991-1992. Inter 99 cohort sampled 6784 men and women in 1999. Health 2006 sampled 4200 men and women from 2006-2008. Follow up is achieved through linkage to the National Cause of Death Register and National Hospital Discharge Register, with endpoint diagnosis based on MORGAM criteria and validation described in 8, 9, 10. Follow up for the cohorts 1, 2, and 3 is completed to December 31st 2010. <http://www.thl.fi/publications/morgam/cohorts/full/denmark/den-gloa.htm>

**Health, Alcohol and Psychosocial factors in Eastern Europe (HAPIEE):** The HAPIEE study comprises four prospective urban population based cohorts from Eastern Europe, including Novosibirsk (Russia), Krakow (Poland), Kaunas (Lithuania) and 7 cities of the Czech Republic (11). Each cohort recruited a random sample of men and women aged 45-69 years at baseline in 2002-05 (2006-08 in Lithuania), stratified by sex and 5-year age group. Participants were selected from population registers (electoral roll list in Russia). Participants completed extensive questionnaire, underwent examination in clinic and provided a blood sample. The sample size (response rates) were 9,360 (61%) in Russia; 10,728 (61%) in Poland; 7,161 (61%) in Lithuania; and 8,857 (55%) in the Czech Republic. Deaths in the cohorts were identified by linkages with national or regional death registers. Non-fatal cardiovascular events were identified via linkage with hospitalisation registers, MONICA registers and by repeated postal follow-up questionnaires validated against GP and hospital records. Follow-up is completed to 2011 for Czech Republic and Lithuania, to 2010 for Russia and to 2009 for Poland. <http://www.ucl.ac.uk/easteurope/hapiee.html>

**Kooperative Gesundheitsforschung in der Region Augsburg (KORA)**: The WHO Multinational Monitoring of Trends and Determinants in Cardiovascular Diseases (MONICA)/ Cooperative Health Research in the Region of Augsburg (KORA) cohorts were randomly selected from representative sample surveys from the city of Augsburg and the less urban Landkreis Augsburg and Landkreis Aichach-Friedberg regions in Bavaria, Southern Germany. List of municipalities and population registers were used as sampling frames for the first and the second stage of two-stage sampling, respectively. The second stage of sampling was stratified by sex and 10-year age group. The Survey 3 (S3) baseline examination (1994-1995) was carried out as part of the WHO MONICA project and consists of 4,480 men and women aged 25-74 with a response rate of 74% (12). The Survey 4 baseline examination (S4) was carried out in 1999-2001 consisting of 3,019 men and women aged 25-74 who were re-examined with repeated measurements collected, in 2006-2008 (F4) with a response rate of 72% (13). S4 and F4 studies and morbidity and mortality follow-ups were conducted in the frame of KORA. Coronary events were identified through the MONICA/KORA Augsburg coronary event registry. Coronary deaths were validated by autopsy reports, death certificates, chart review from the last treating physician. Cases with self-reported incident diabetes were validated by the treating physician and medical records documenting use of antidiabetic medication. Self-reported cases of incident stroke were validated by medical records. Mortality follow-up until 2009 was conducted through national death registers. <http://www.thl.fi/publications/morgam/cohorts/full/germany/ger-auga.htm>

**Moli-Sani Project:** The cohort of the Moli-Sani Project was recruited in the Molise region from city hall registries by a multistage sampling. First, townships were sampled in major areas by cluster sampling; then, within each township, participants aged 35 years or over were selected by simple random sampling. Exclusion criteria were pregnancy at the time of recruitment, lack of in understanding, current multiple trauma or coma, or refusal to sign the informed consent. A total of 24,325 men (47%) and women (53%) over the age of 35 were examined at baseline from 2005 to 2010. Participation was 70%. The cohort was followed-up for a median of 4.2 years (maximum 6.5 years) at December 2011 and will be followed-up every 5 years (14). Follow up is achieved through record linkage to national mortality registries and hospital discharge registers, validation of events was achieved through hospital record linkage and doctors medical records using updated MORGAM criteria. <http://www.moli-sani.org/>

**MONICA Brianza Study**: The MONICA-Brianza Cohort Study is a prospective observational study of three cohorts of 25-64 years old residents in Brianza, a highly-industrialized area located between Milan and the Swiss border, Northern Italy. Gender- and 10-year age stratified samples were randomly drawn in 1986, 1990 and 1993, and cardiovascular risk factors were investigated at baseline following the procedures of the WHO MONICA Project (21). The overall participation rate was 69%. For all subjects whole blood and serum samples were stored in a biobank. The protocol was approved by the Monza Hospital Ethical Committee. Baseline examinations were carried out in 1986-87 for cohort 1 of N=1,659 response rate was 69%, Cohort 2 was collected in 1989-90 (N=1599), response rate was 67% and cohort 3 in 1993-1994 (N=1674), response rate was 69%. Study participants were enrolled and followed up for first coronary or stroke events, fatal and non-fatal (22), up to the end of 2008, for a median of 15 years. [http://www4.uninsubria.it/on-line/home/naviga-per-tema/ricerca-scientifica/centri-di-ricerca/centro-di-ricerca-in-epidemiologia-e-medicina-preventiva-epimed.html](https://webmail.uke.de/owa/redir.aspx?C=CSmewM6SiUaC4NHH3KQGn5d4-VLl0NAIKBwKOfHzDkAZJcWyNdE-HrOnh5eedrMPt5bpjbvEzJw.&URL=http%3a%2f%2fwww4.uninsubria.it%2fon-line%2fhome%2fnaviga-per-tema%2fricerca-scientifica%2fcentri-di-ricerca%2fcentro-di-ricerca-in-epidemiologia-e-medicina-preventiva-epimed.html)

**MONICA-Catalonia Study:** The Catalonia Study consists of two cohorts sampled from representative surveys from the central area of Catalonia and part of the metropolitan area of Barcelona, Spain. The first stage drew a random sample of individuals from nine municipalities with probability proportional to population size. In the second stage, a random sample of men and women from the Municipal population registries were used as the sampling frame, from each municipal population register, a random sample stratified by sex and ten-year age groups, 25-64 years, of fixed number was selected. Baseline examinations were carried out in 1986-1988 for cohort 1 (Round 1) of N=2,571, response rate was 74%. Cohort 2 (Round 1) was collected in 1990-1992 (N=2,936), response rate was 67%. Participants were mainly from the Industrial and services economy (17, 18, 19). All subjects gave informed consent. The project was approved by the Institute of Health Studies steering committee. Follow up until 1997 for Cohort 1 and until 1999 for Cohort 2 was achieved through follow up questionnaires and record linkage with MONICA registers, national mortality index register and hospital discharge registers.

**MONICA Friuli:** The Friuli population cohorts were collected as part of the WHO MONICA surveys. The Friuli area covers three provinces of north-east Italy (Friuli-Venezia-Giulia) recruited using a single stage sampling frame from the official Regional Health Roll stratified by Health Unit (combination of municipalities covering 40,000 inhabitants), sex and 5-year age group. Four cohorts were recruited, the BiomarCaRE project will focus on Cohort 3 collected in 1994 (participation rate, 71%) consisting of men and women aged 25-64 years and cohort 4 collected in 1995-96, (participation rate, 90% consisting of men and women aged 45-64 years. Follow up through registry linkage is available until 1998 collected through the framework of the Progetto Cuore. [http://www.cuore.iss.it/eng/assessment/procedures.asp](https://ex2k7.qub.ac.uk/OWA/redir.aspx?C=yKuB25KnakeLsDzD3CxULRH1k6Qzr9BI074qTYYGAZq1MDpi9cc3C_0BWC_pyboqk7p8gtGOrPc.&URL=http%3a%2f%2fwww.cuore.iss.it%2feng%2fassessment%2fprocedures.asp)

**The Northern Sweden MONICA Study:** The Northern Sweden MONICA study covered the two northernmost counties of Sweden, i.e. Norrbotten and Västerbotten with altogether 510,000 inhabitants. Population surveys were performed in 1986, 1990, 1994, 1999, 2004 and 2009, with altogether 10,517 unique participants (20). On the first two occasions, 2,000 persons aged 25 to 64 years were randomly selected, and in the last three surveys, the upper age limit was extended to 74 years and 2,500 individuals were invited. A stratified randomized selection procedure by age and sex (250 persons in each sex/10-year age stratum) has been used. The participation rate was 69-81%. Detailed analyses of non-participants have been performed. In 1999, all people invited to any of the three previous population surveys were re-invited for repeated measurements to be collected. Incident cardiovascular events (myocardial infarction and stroke) occurring in the region between 1985 and 2010 and below the age of 75 were collected and validated according to MONICA criteria by two event registers whose accuracy and validity have been tested against national registers (21). Follow-up is available for all cohorts until December 2011 for mortality and non- fatal coronary, stroke, chronic heart failure, atrial fibrillation, cancer and diabetes events. Coronary and stroke events below the age of 75 validated applying the MONICA diagnostic criteria, and diabetes according to careful case review (22). <http://www9.umu.se/phmed/medicin/monica/index.php?content=startsida>, <http://www.thl.fi/publications/morgam/cohorts/full/sweden/swe-nswa.htm>

**Prospective Epidemiological Study of Myocardial Infarction (PRIME) Study:** The PRIME study examined the classic and putative cardiovascular risk factors to explain the large difference in heart disease incidence between Ireland and France (23). The study includes four cohorts of men aged 50-59; from Belfast, Northern Ireland (N=2,745) and Lille (N=2,633), Toulouse (N=2,610) and Strasbourg (N=2,612) in France. Baseline examinations took place in 1990-1993 and targeted cohorts which had broadly similar social class structures to the background population, initially sampling from industries and various employment groups, employment groups with more than 10% of their workforce of foreign origin were excluded. Follow up until 2004 (Toulouse, Strasbourg and Lille) and until 2012 (Belfast) was achieved through annual follow up questionnaires with verification against national death registers, medical records, hospital discharge diagnoses. Endpoints were validated by expert medical committee. <http://www.thl.fi/publications/morgam/cohorts/full/uk/unk-bela.htm>; <http://www.thl.fi/publications/morgam/cohorts/full/france/fra-lila.htm>; <http://www.thl.fi/publications/morgam/cohorts/full/france/fra-stra.htm>; <http://www.thl.fi/publications/morgam/cohorts/full/france/fra-toua.htm>

**Scottish Heart Health Extended Cohort (SHHEC):** This consists of two overlapping studies which share a common protocol and methods: the Scottish Heart Health Study randomly recruited men and women aged 40-59 across 22 Scottish districts in 1984-1987; Scottish MONICA similarly recruited men and women aged 25-64 in Edinburgh and North Glasgow in 1986, and in North Glasgow again in 1989, 1992 (to 74), and in 1995 as part of the WHO MONICA Project. They are now combined as one cohort, although length of follow-up, currently to the end of 2009, inevitably varies in the different components. Follow up is achieved through flagging for death certificates at the National Health Service Death Register and through the Scottish Record Linkage scheme for deaths and hospital discharge records run by Information Services Scotland, which works on probability matching (24). These diagnoses are no longer validated from case notes now that endpoint numbers run into thousands as the cohort ages, but they are allocated to MORGAM categories. Risk factor and endpoint data were used to produce the ASSIGN cardiovascular risk score (25). See. <http://www.thl.fi/publications/morgam/cohorts/full/uk/unk-sco.htm>

**Study of Health in Pomerania (SHIP):** The SHIP study is an established population-based project conducted in Northeast Germany. The study aims to assess prevalence and incidence of common risk factors, subclinical disorders and clinical diseases and to investigate associations and interactions among them using comprehensive medical assessments (26). The first SHIP cohort was recruited between 1997 and 2001 and included 4,308 individuals at baseline (SHIP-0, 20-70 years, response 68.8%), 3,300 after five years (SHIP-1) and 2,333 after 11 years (SHIP-2). In parallel to SHIP-2, baseline examinations of a second, independent cohort (SHIP-TREND) were conducted in 4,420 participants (20-79 years, response 50.3%). SHIP is one of the population-based projects with very comprehensive examinations including interviews, cardio-metabolic ultrasound exams, cardiopulmonary exercise tests and whole-body magnetic resonance imaging in a general population setting. In addition to the examination follow-ups, information on fatal and non-fatal disease is collected on a regular basis. Mortality follow-ups are conducted semi-annually by record linkage with data bases of the regional population registry. Causes of death are defined from the official death documents provided by regional health authorities. Active follow-ups for non-fatal diseases are performed biannually and by interviews during follow-up examinations every five years. Self-reported information is validated by GP’s and using databases of the regional Association of SHI Physicians. [http://www.medizin.uni-greifswald.de/cm/fv/ship.html](https://ex2k7.qub.ac.uk/OWA/redir.aspx?C=DjaCEmcttEeAM9Q7e342WwsOemrPltBIAaGIxNopT3bPhlFahxdKpYPY70Ud1nsEUmW7TmUMEfs.&URL=http%3a%2f%2fwww.medizin.uni-greifswald.de%2fcm%2ffv%2fship.html) <http://www.medizin.uni-greifswald.de/cm/fv/ship.html>

**The Rome Study (Malattie Aterosclerotiche Istituto Superiore di Sanità (MATISS), (Italy MONICA)):** The MATISS study started in 1984 as DiSCo - DIstretto Sezze controllo COmunitario - designed as demonstration project of non-communicable diseases in Central Italy. Four municipalities were involved, three at community treatment and one at control. Baseline (1984) and 4-year examinations were used to evaluate in a random sample of the general population community treatment and compared with control area (27). The project followed as prospective, observational study (MATISS). In 1993-96 the cohorts were re-examined (2,519 persons) and a new random sample, stratified by age and sex, was enrolled from the residence registry (1,970 persons examined, participation rate 60%). Follow-up is available for all cohorts until December 2004 for mortality and non- fatal coronary and stroke events, validated applying the MONICA diagnostic criteria.

**The Tromsø Study:** The Tromsø Study is a prospective repeated population-based health survey of men and women aged 20-97 years in Northern Norway (28). Specific age groups in the municipality were invited to the different surveys and over time this enabled collection of repeated risk factor measurements in many subjects. The 3^rd^ Tromsø Study survey (Tromsø) was carried out in 1986-87 and those invited were all men in the 20-61 age group, all women in the 20-56 age group, a randomly selected 10% sample from the 12-19 age group (born 1967-1974) and a subsample who were included in a family intervention study. A total of 21,826 participated, 75% of the invited. Of these, data on all men and women aged 20-59 years will be included in the BiomarCaRE study (n= 20,300), including blood samples from Tromsø 3 and also from the subsequent Tromsø 4 survey (participation rate 77%, n=27,158) which contains repeated measurements from Tromsø 3. The cohort is being followed up with registration of incident myocardial infarction, stroke (ischemic, hemorrhagic, subarachnoid haemorrhage and unclassifiable), diabetes, atrial fibrillation and cause-specific death. Cases of incident events were identified by linkage to the diagnosis registry at the University Hospital of North Norway (the only hospital in the region) and to the National Causes of Death Registry. Validation of hospitalized and out-of hospital events was performed by an independent endpoint committee and based on data from hospital and out-of hospital journals, autopsy records, and death certificates. Slightly modified World Health Organization MONICA/MORGAM criteria for myocardial infarction (29) and stroke (30) were used. Follow-up is completed to December 31st 2010. <http://tromsoundersokelsen.uit.no/tromso/>

**Disease Cohorts**

**Athero*Gene*:** The Athero*Gene* study was conducted at the Department of Medicine II of the University Medical Center Mainz and the Federal Armed Forces Central Hospital Koblenz in Germany from 1996 to 2004 (31). The major goal of the Athero*Gene* study was to assess risk prediction across a wide range of coronary artery disease phenotypes with particular emphasis on the role of genetics. The major endpoint in this study was cardiovascular death and non-fatal myocardial infarction among other cardiovascular outcomes. Inclusion criteria for this study were prevalent coronary artery disease with angiographical diagnosis of a 30% or higher stenosis in a major coronary artery. Exclusion criteria were evidence of hemodynamically significant valvular heart disease, surgery or trauma within the previous month, known cardiomyopathy, malignancies, febrile conditions, chronic inflammatory diseases, renal failure (increased creatinine >2.1 mg/dL) or use of oral anticoagulant therapy within the previous four weeks. Patients presenting with solely elevated C-reactive protein concentrations were not excluded from the study cohort. Fasting blood was drawn prior to catheterization in the cath lab and processed immediately.

**Advantageous Predictors of Acute Coronary Syndromes Evaluation (APACE) Study:** Consecutive patients who presented to the emergency department with symptoms suggestive of AMI between April 2006 and June 2009 were enrolled in this prospective, international, multi-center study as described previously (32). Blood samples were collected in serum or EDTA plasma tubes at the time of presentation and additional samples were obtained 1, 2, 3, and 6 hours after presentation. Samples were centrifuged and aliquots were stored at -80°C. The study was approved by the local ethics committee and each patient gave written informed consent. <http://www.dyspnea.ch/apace.htm>

**Langzeiterfolge der Kardiologischen Anschlussheilbehandlung (KAROLA):** KAROLA is an observational, prospective cohort study in which 1,206 patients with coronary heart disease (CHD) aged 30-70 years participating in an in-hospital rehabilitation program between January 1999 and May 2000 in two co-operating clinics (Schwabenland-Klinik, Isny and Klinik am Südpark, Bad Nauheim, Germany) were enrolled. Only patients who were admitted within 3 months after their first acute event or coronary artery revascularization were included. Baseline data included detailed information from medical records, medical exams and patient questionnaires. An active follow-up has been conducted 1, 3 and 4.5, 6, 8, 10 and 13 years later (33, 34). Beside life style factors, quality of life and other patient reported information obtained by contacting the patient, the occurrence of secondary cardiovascular disease events was evaluated by contacting the primary care physicians. If a subject died during follow-up, the death certificate was obtained from local Public Health departments and the main cause of death was coded according to the International Classification of Diseases. Follow-up is ongoing. <http://www.dkfz.de/de/klinepi/Projekte/karola-studie.htm>

**Study for evaluation of newly onset chest pain and rapid diagnosis of myocardial necrosis (steno*Cardia*):** Patients with acute chest pain presenting consecutively at the chest pain unit of the Johannes Gutenberg-University Medical Centre Mainz between January 2007 and December 2008 were enrolled in this all-comers prospective biomarker assessment registry (35, as described earlier (36). Blood samples were obtained on admission and after 3 and 6 hours. Routine laboratory parameters including C-reactive protein were measured immediately after blood withdrawal by standardized methods. Additionally, EDTA plasma and serum samples were collected at each time point, centrifuged, aliquoted and stored at -80°C. The study was approved by the local ethics committees. Participation was voluntary; each patient gave written, informed consent.

**UCSC-ACS: prospective cohort under enrollment at the Cardiology Institute of the Catholic University of Rome:** The major goal of the UCSC-ACS study is to provide a large and contemporary cohort of patients hospitalized for acute ischemic heart disease, with a prevalence of NSTEMI-UA. The subjects are treated with invasive procedures or cardiac surgery, unless contraindicated and cover a large spectrum of ages and conditions. All patients will be followed for at least 6 months and up to one year for all relevant events (new hospitalization for any cause, ACS, heart failure or death) will be recorded. Exclusion criteria are severe comorbidities limiting life expectancy at less than one year, active cancer, sepsis or chronic and acute inflammatory diseases. Fasting blood is taken within 12 hours from hospital admission for UA/NSTEMI and 30 min for STEMI, which is divided into 100 microliter aliquots and immediately stored at -80 degrees (37). [www.rm.unicatt.it](http://www.rm.unicatt.it)

**Clinical Trials**

**Integrated Biomarker and Imaging Study-2 (IBIS-2):** IBIS-2 (Study 026) was a multi-center, randomized, double blind, placebo-controlled, parallel group study designed to examine the effects of once daily Darapladib Enteric Coated Tablet 160 mg (n = 172) on intermediate cardiovascular endpoints compared to placebo (n = 151) (38). The study evaluated intravascular ultrasound (IVUS)-based imaging parameters, circulating biomarkers, endothelial function, safety and tolerability over the 1-year treatment duration in subjects from 10 countries in Europe (Denmark, Netherlands, Germany, Switzerland, Poland, Austria, Spain, Norway, Belgium, Czech Republic). By design, the study included approximately 50% of subjects with acute coronary syndrome (ACS), either ST-elevation Myocardial Infarction (STEMI) or Non-STEMI, or non-ACS patients with coronary heart disease. The mean age of subjects was 58 years (range, 33 to 82 years), and the majority of participants were white (98%) and male (82%).

**The Long-Term Intervention with Pravastatin in Ischaemic Disease (LIPID) Study:** the LIPID Study was a randomised, double-blind, placebo-controlled parallel group study conducted in 9,014 patients recruited from 87 centres in Australia and New Zealand (39). It was designed to examine the effects of 40mg pravastatin o.d on cardiovascular outcomes in patients who were stable after myocardial infarction or hospitalisation for unstable angina 3-36 months previously. Patients had baseline total cholesterol of 4.0-7.0mmol/L and triglycerides <5mmol/L and at baseline a high percentage received other evidence-based therapies for coronary heart disease. Over a mean follow-up of 6.0 years there was a highly significant reduction in coronary heart disease mortality, the primary study endpoint, total mortality and all other prespecified cardiovascular end-points.

<http://www.ctc.usyd.edu.au/our-research/clinical-trials/diabetes-and-cardiovascular/lipid.aspx>

**The Justification for the Use of Statins in Primary Prevention: An Intervention Trial Evaluating Rosuvastatin (JUPITER) trial:** JUPITER is a completed, randomized, double-blind, placebo-controlled trial of rosuvastatin 20 mg daily in the prevention of cardiovascular disease. Men who were at least 50 years old and women at least 60 years old were eligible for the trial if they did not have preexisting cardiovascular disease or diabetes, and had a low density lipoprotein cholesterol < 130 mg/dL and C-reactive protein ≥ 2.0 mg/L. JUPITER enrolled and randomized 17,802 subjects who were then followed for a median of 1.9 years (40, 41).

<http://www.brighamandwomens.org/research/depts/medicine/preventive_medicine/research_areas/jupiter_trial.aspx>

**The Telmisartan Randomised Assessment Study in ACE intolerant subjects with cardiovascular disease (TRANSCEND) Trial:** In the TRANSCEND Trial, eligible patients were entered into a single blind run-in involving placebo daily for a week followed by 2 weeks of telmisartan 80 mg. At the end of this run-in period, patients were randomised in a one to one ratio by use of a central automated randomisation system to receive telmisartan (80 mg/day) or placebo. Patients were assessed at follow-up visits scheduled at 6 weeks and 6 months, and then every 6 months The aim was to investigate whether an angiotensin receptor blocker—telmisartan—when given long term, reduces cardiovascular death, myocardial infarction, stroke, or hospitalisation for heart failure in patients with cardiovascular disease or high-risk diabetes and without heart failure, who are intolerant of ACE inhibitors, compared with placebo (42). <http://www.trialresultscenter.org/study8595-TRANSCEND.htm>

**The Women's Health (WHS) Study**: The WHS is a completed, randomized, double-blind, placebo controlled 2x2 factorial trial of vitamin E and aspirin in the prevention of cardiovascular disease and cancer in nearly 39,876 initially healthy female health professionals aged 45 years and older in the United States (43, 44). Enrolment began in 1993, and the randomized trial portion of the study was completed in March 2004. Participants were invited to continue in an observational follow up cohort. In total, 28,345 women provided blood samples at baseline for analysis. <http://whs.bwh.harvard.edu/publications2.html>

**Funding and Acknowledgements of Cohorts**

The MORGAM Project has been funded by THL, through several grants from the EU, the latest ones being from the Seventh Framework Programme (FP7/2007-2013) under grant agreements No. HEALTH-F2-2011-278913, BiomarCaRE), FP7/2007-2013 [HEALTH-F4-2007-2014113, ENGAGE and HEALTH-F3-2010-242244, CHANCES], and the Medical Research Council London [G0601463, No 80983: Serum Biomarkers in the MORGAM Populations]. These have supported central coordination, workshops and the activities of the MORGAM Data Centre, at THL in Helsinki, Finland.

**General Population based cohorts:**

Alpha-Tocopherol, Beta-Carotene Cancer Prevention (ATBC) Study was supported by Public Health Service contracts N01-CN-45165, N01-RC-45035, N01-RC-37004, and HHSN261201000006C from the U.S. National Cancer Institute, National Institutes of Health, Department of Health and Human Services, and the Intramural Program of the U.S. National Cancer Institute.

Caerphilly Prospective Study (CaPs); This study was supported by a Project Grant from the British Heart Foundation (PG/09/002/26056).

Estonian Genome Center of the University of Tartu (EGCUT) – The Estonian Biobank: This work was supported by the Targeted Financing from the Estonian Ministry of Science and Education (SF0180142s08) and by the grant from the Development Fund of the University of Tartu (SP1GVARENG).

FINRISK: The FINRISK surveys were mainly funded by budgetary funds of THL. Additional funding has been obtained from numerous non-profit foundations. Dr. Salomaa (PI) has been supported by the Finnish Foundation for Cardiovascular Research and the Academy of Finland (grant number 139635).

The Gutenberg Health Study is funded through the government of Rheinland-Pfalz (No. AZ 961-386261/733), the research program “Wissen schafft Zukunft” and the “Schwerpunkt Vaskuläre Prävention” of the University Medical Center Mainz and its contract with Boehringer Ingelheim and PHILIPS medical systems, including an unrestricted grant for the Gutenberg Health Study. This project was further supported by the MAIFOR program of the University Medical Center Mainz and the National Genome Network “NGFNplus” by the federal Ministry of Education and Research, Germany (No. 01GS0833 and 01GS0831, projects A3/D1).

The Glostrup Study cohorts at the Research Centre for Prevention and Health were established over a period of 45 years and have been funded by numerous sources which have been acknowledged, where appropriate, in the original articles.

Health, Alcohol and Psychosocial factors in Eastern Europe HAPIEE: This study is funded by grants from the Wellcome Trust (grant no.s 064947/ Z/01/Z and 081081/Z/06/Z), the US National Institute on Aging (grant no. 1R01 AG23522) and the MacArthur Foundation (Health and Social Upheaval network). We would like to thank researchers, interviewers and participants in Novosibirsk, Krakow, Kaunas, Havířov/Karviná, Jihlava, Ústí nad Labem, Liberec, Hradec Králové, and Kromĕříz.

The KORA research platform (KORA, Cooperative Health Research in the Region of Augsburg) was initiated and financed by the Helmholtz Zentrum München – German Research Center for Environmental Health, which is funded by the German Federal Ministry of Education and Research and by the State of Bavaria. Furthermore, KORA research was supported within the Munich Center of Health Sciences (MC Health), Ludwig-Maximilians-Universität, as part of LMUinnovativ. The KORA-Study Group consists of A. Peters, J. Heinrich, R. Holle, R. Leidl, C. Meisinger, K. Strauch, and their co-workers, who are responsible for the design and conduct of the KORA studies.

The MOLI-SANI Project was partially supported by research grants from Pfizer Foundation (Rome, Italy), the Italian Ministry of University and Research (MIUR, Rome, Italy)–Programma Triennale di Ricerca, Decreto n.1588 and Instrumentation Laboratory, Milan, Italy

The MONICA Brianza Study was funded by the Regione Lombardia Health Administration grant number 10800/2009, as part of the Osservatorio Epidemiologico Cardiovascolare Regionale Lombardo - Progetto CAMUNI III and it is part of the CUORE Project. G. Veronesi, M Ferrario, G. Cesana, B. Brambilla, F. Gianfagna; S. Signorini.

The MONICA-Catalonia Study and follow-up were funded by the Institute of Health Studies, Department of Health of Catalonia. Biobank facilities and support are provided by the Catalan Institute of Cardiovascular Sciences.

The MONICA Friuli Project was initiated by the late Prof. G.A. Feruglio, a man of great vision, and it was funded with grants from the Regione Friuli Health Administration. D. Vanuzzo.

The Northern Sweden MONICA Study was supported by grants from Norrbotten and Västerbotten County Councils, the Swedish Research Council (MFR), the Joint Committee of Northern Sweden Health Care Region (Visare Norr), the Heart and Chest Foundation, the Stroke fund, King Gustaf V’s and Queen Victoria’s Foundation, the Vårdal Foundation and the Social Sciences Research Council.

Prospective Epidemiological Study of Myocardial Infarction PRIME Study: We thank the following organizations which allowed the recruitment of the PRIME subjects: the Health screening centres organized by the Social Security of Lille (Institut Pasteur), Strasbourg, Toulouse and Tourcoing; Occupational Medicine Services of Haute- Garonne, of the Urban Community of Strasbourg; the Association Inter-entreprises des Services Médicaux du Travail de Lille et environs; the Comité pour le Développement de la Médecine du Travail; the Mutuelle Générale des PTT du Bas-Rhin; the Laboratoire d’Analyses de l’Institut de Chimie Biologique de la Faculté de Médecine de Strasbourg; the Department of Health (NI) and the Northern Ireland Chest Heart and Stroke Association. We also thank the members of the event validation Committees: Pr L. Guize, Dr C. Morrison, Dr M-T. Guillanneuf, Pr M. Giroud; and the Alliance Partnership Programme for its financial support. The PRIME Study was supported by grants from INSERM, and the Merck, Sharp and Dohme-Chibret Laboratory. PRIME Belfast received funding from HSC Research and Development Office and the Medical Research Council London [G0601463, No 80983: Serum Biomarkers in the MORGAM Populations].

The Scottish Heart Health Extended Cohort (SHHEC) was funded by the Scottish Health Department Chief Scientist Organization; British Heart Foundation; FP Fleming Trust.

The Study of Health in Pomerania (SHIP) is part of the Community Medicine Research net (CMR) of the University of Greifswald, Germany, which is funded by the Ministry of Cultural Affairs and the Social Ministry of the Federal State of Mecklenburg-West Pomerania. The CMR encompasses several research projects which share data from several population-based projects [http://community-medicine.de](https://ex2k7.qub.ac.uk/OWA/redir.aspx?C=DjaCEmcttEeAM9Q7e342WwsOemrPltBIAaGIxNopT3bPhlFahxdKpYPY70Ud1nsEUmW7TmUMEfs.&URL=http%3a%2f%2fcommunity-medicine.de)).

The Rome MATISS Study (Malattie Aterosclerotiche Istituto Superiore di Sanità) is part of the CUORE Project - Epidemiology and Prevention of Cardiovascular Disease, funded by the Italian Ministry of Health and coordinated by the Unit of Epidemiology of Cardiovascular Disease of the National Centre for Epidemiology, Surveillance and Health Promotion, IstitutoSuperiore di Sanita, Rome, Italy. L. Palmieri, C Donfancesco, S Giampaoli.

The Tromso Study is mainly funded by the University of Tromsø, with substantial contributions from the Research Council of Norway, the National Screening Services, the Northern Norway Regional Health Authority, the Norwegian Council on Cardiovascular Diseases and the Norwegian EXTRA Foundation for Health and Rehabilitation.

**Disease cohorts:**

AtheroGene. The AtheroGene study is supported by a grant of the “Stiftung Rheinland-Pfalz für Innovation”, Ministry of Science and Education (AZ 15202-386261/545), Mainz, Germany, and by a grant from the Fondation de France (no. 2002004994).

The APACE study was supported by research grants from Swiss National Science Foundation, Swiss Heart Foundation, the Department of Internal Medicine, University Hospital Basel, the University Basel, Abbott, Brahms, Roche, and Siemens.

KAROLA; The study was supported in parts by the German Federal Ministry of Education and Research (#01GD9820/0) and the Pitzer Foundation (Bad Nauheim, Germany).

The stenoCardia Study was supported by the research programs “Wissen schafft Zukunft”, “Schwerpunkt Vaskulaere Praevention” and “MAIFOR 2010” of the Medical University Center Mainz for the stenoCardia study. In addition, funding was provided by unrestricted grants of the Brahms AG and Abbott Diagnostics.

**Clinical Trials:**

IBIS-2. The IBIS-2 Trial was a clinical trial fully sponsored by GlaxoSmithKline (GSK).

The LIPID study was funded by Bristol-Myers Squibb Pty Ltd and conducted under the auspices of the National Heart Foundation of Australia.

JUPITER was funded by AstraZeneca, who collected the trial data and monitored the study sites but had no role in the conduct of these analyses or in drafting this report

The Women’s Health Study (WHS) was supported by grants HL-043851, HL-080467, and HL- 099355 from the National Heart, Lung, and Blood Institute and CA-047988 from the National Cancer Institute and the Donald W. Reynolds Foundation.

**Description of established and emerging, literature-based biomarkers**

**Established, literature-based markers**

***Cardiac troponins***

Troponins are sensitive markers of myocardial injury independent of the primary cause. Troponins play a pivotal role in the diagnosis of acute coronary syndromes (ACS) and the routine management of these patients for almost 15 years. The introduction of high-sensitive (hs) assays which are ten times more sensitive than previous tests enable an even earlier detection of myocardial ischemia at very low levels of approximately 3ng/l. In addition to the undisputable diagnostic value of troponins in the clinical setting there is increasing evidence for a prognostic value of even small increases of this biomarker with fairly strong associations with future heart failure, myocardial infarction, and death (45). There are several high-sensitive or even ultra-sensitive assays available, most of which have not been tested in large populations so far. Initial studies suggest that, based on kinetics and other properties there may be differences between various troponins that could have clinical implications. Thus, further research is required to adequately test available high sensitive assays for their prognostic value in diverse populations (46, 47, 48, 49, 50, 51)

***Natriuretic peptides***

Natriuretic peptides represent a group of biomarkers that are secreted after hemodynamic stress by the myocardium during various conditions like myocardial infarction, hypertension, valvular diseases, pulmonary embolism, atrial fibrillation, etc. There are several natriuretic peptides available in particular brain natriuretic peptide (BNP) and N-Terminal proBNP which clinically represent the two most relevant molecules. In addition, there is midregional proANP (MR-proANP) and adrenomedullin which have not been studied extensively so far. Determination of BNP or NT-proBNP already represent an integral part of congestive heart failure (CHF) guidelines and both have been proven to be of value in the differential diagnosis of dyspnea. In addition, ongoing research is related to their potential to guide therapy together with clinical symptoms. There is also increasing data, both from population-based studies (52) as well as in patients with manifest disease (53) and in patients with ACS and post-ACS (54) that clearly document their predictive value for recurrent cardiovascular (CV) events. In addition, formal meta-analyses are in support of such a role (55). From a clinical standpoint, BNP and NT-proBNP do carry similar prognostic capabilities. However, there might be differences with regard to potential confounding variables that may affect the absolute level of these hormones, in particular renal function. Yet, there is still a need for further evaluation of these markers of hemodynamic stress in diverse populations with regard to various fatal and non-fatal CV outcomes (56,57, 58, 59).

***C-reactive protein***

C-reactive protein (CRP) represents an exquisitely sensitive marker of systemic inflammation. It is produced by the liver, mainly upon stimulation by various cytokines and represents an unspecific marker of acute or ongoing inflammation in any part of the body. During the last 15 years it has been studied extensively for its potential to improve prediction of cardiovascular events in various settings. Several meta-analyses have clearly established a positive association between elevated levels of hsCRP and various cardiovascular outcomes (60) and a consistent, albeit small improvement in risk prediction could be demonstrated if hsCRP was added to traditional risk factors including HDL cholesterol (61). Based on such data it has been recommended by the 2010 ACCF/AHA Guidelines to be incorporated in the diagnostic work-up of subjects with intermediate risk or in those with JUPITER inclusion criteria (62) with a similar statement coming from the United States Public Health Service (63). It is already in the Guidelines for screening patients at cardiovascular risk by the Canadian Cardiovascular Society (64). Yet it still represents a controversial issue based on its variability under various conditions due to the fact that it is completely unspecific. However, it is the only biomarker so far, that has been prospectively tested in a clinical trial, JUPITER, to identify a high-risk group of subjects for aggressive treatment with statins that would not have qualified otherwise for this intervention (65). Recently, data from the EPIC Norfolk Study in more than 8,000 subjects have confirmed the clinical implications of JUPITER (66). There is a large number of further inflammatory biomarkers e.g. various cytokines like TNF-α, IL-1, IL-6, IL-18, chemokines like MCP-1, cellular adhesion molecules like sICAM-1 and sVCAM-1, e-Selectin, ADMA, and markers possibly more closely related to plaque destabilization and plaque rupture, namely oxLDL, Lp-PLA2, GPX1, MPO, MMPs, PlGF, PAPP-A, neopterin, and soluble CD40 ligand. However, all of these markers do lack such a broad basis of evidence as it has been accumulated for hsCRP and many of them, because of analytical issues, do not seem to be suitable as clinically relevant biomarkers. However, several of these molecules have already been identified as potential targets for intervention and are currently being tested in clinical trials like i.e. Lp-PLA_2_ in STABILITY and SOLID.

***Markers of lipid metabolism (LDL cholesterol, HDL cholesterol, triglycerides, lipoprotein(a) (Lp(a)), apoA1 and apoB100)***

Total cholesterol, LDL cholesterol (LDL-C), HDL cholesterol (HDL-C) and triglycerides represent routine parameters reflecting lipid metabolism and are used in daily practice. In particular, LDL-C is consistently found to be strongly associated with future cardiovascular outcomes in large population-based studies. It serves to identify patients at cardiovascular risk and also represents the most important marker to target lipid-lowering therapy based on various guidelines (67, 68). Thus, in all prediction models adjustment for at least total cholesterol and HDL-C or LDL-C and HDL-C has to be done. The additional value of apolipoproteins like apoA1 and apoB100 for risk prediction is still being debated. Although the determination of these two biomarkers offers several advantages in terms of analytical issues over total/ LDL-C and HDL-C, a recent analysis could not clearly document superior risk prediction based on these molecules (69). The same applies to lipoprotein-associated phospholipase A2 (Lp-PLA_2_) and lipoprotein(a). Further large-scale studies put forward by the Emerging Risk Factors Collaboration (70) concluded that the lipid assessment in vascular disease might be simplified by measurement of either total and HDL-C levels or apolipoproteins without the need to fast and without regard to triglycerides. This represents clinically important information for screening subjects at risk.

Despite such data, the potential contribution of elevated triglycerides to risk prediction is still under discussion. In earlier epidemiological studies, triglycerides have not been measured, thus, the database is much smaller compared to total and HDL-C. However, several recent studies have clearly documented a prognostic role of even non-fasting triglycerides in addition to total/ LDL-C (71, 72, 73), and triglycerides may be even causally related to coronary heart disease (CHD) (74). Elevated triglycerides together with HDL-C are particularly important in the context of the metabolic syndrome and/or manifest diabetes.

With regard to HDL-C there is a broad epidemiological basis that clearly demonstrates a strong inverse association between increasing levels of HDL-C and cardiovascular outcomes (70, 75. In addition, it has been shown to represent a strong predictor of progression in patients with manifest CHD (76). Therefore, HDL-C has been recently introduced into the ESC Guidelines as an additional lipid marker to be considered beyond total cholesterol. However, considerable uncertainty still exists with regard to HDL-C as a potential therapeutic target after the failure of various HDL-C increasing trials like AIM-HIGH (77) or HPS-THRIVE (78). Recently, the European Atherosclerosis Society has issued a consensus statement regarding triglycerides and HDL-C in patient management (79).

Finally, Lp(a), a structurally and functionally unique lipoprotein consisting of a glycoprotein apolipoprotein(a) which is covalently linked to LDL-C needs to be mentioned. Lp(a) is highly heritable (80, 81), and has been discovered in 1963 by Berg. Since then many prospective epidemiological studies have shown an increased risk of Lp(a) above 30 mg/dl for various cardiovascular outcomes. However, in the majority of these studies, the contribution of Lp(a) was modest, in particular in those subjects with normal LDL-C. The main excess risk was seen in those subjects with high LDL-C and those who in addition also had high Lp(a). For a long time, the knowledge base was relatively controversial which may be attributed to the fact that Lp(a) particles are heterogeneous. Also, there is fairly poor agreement among Lp(a) levels obtained by different assays. So, analytical problems in addition to the above mentioned biological variation may have contributed to the inconsistent knowledge base. Of note, elevated Lp(a) levels are fairly prevalent in the general population with about 20% of individuals exhibiting levels of > 50 mg/dl. Despite such evidence from prospective studies showing an association between elevated levels of Lp(a) and various cardiovascular endpoints, the physiological and vascular effects of the particle remain uncertain. Yet it has been shown to enter the arterial intima of humans in vitro and animal studies have reported that Lp(a) can promote thrombosis, inflammation, and foam cell formation. Most importantly, it is well known that increased levels of Lp(a) in the circulation interfere with the plasminogen receptor and thus may be pro-thrombogenic. A paucity of data is available for diabetes. Recently, large meta-analyses (82) clearly established a continuous independent yet modest association of Lp(a) with risk of CHD and stroke. Based on such circumstantial evidence again the European Atherosclerosis Society has issued a consensus report on the current status of Lp(a) as a cardiovascular risk factor and concluded that screening for Lp(a) in those at intermediate or high CVD/CHD risk is needed and a desirable level should be < 50 mg/dl as a function of global cardiovascular risk (83). Because of the above mentioned analytical problems based on different assays available, further large-scale evaluation of Lp(a) in the risk prediction of cardiovascular disease is needed.

***Biomarkers of renal function***

Serum creatinine levels as a marker of renal function have been used in clinical practice for many years. There are several assays available for measurement. Impaired renal function as measured by elevated serum creatinine concentrations have been clearly shown to be associated with increased cardiovascular risk. Serum creatinine together with age and sex has been used in several formulas to estimate glomerular filtration rate like the Cockcroft and Gault formula, MDRD, and CKD-EPI (84). However, in the intermediate range between 60 and 90 ml/min creatinine is not very effective in assessing renal function. Thus, recently, cystatin C, a new biomarker has become of interest. Cystatin C is a member of the cysteine protease inhibitor family that regulates proteases secreted from lysosomes and inhibits extracellular matrix breakdown. It is produced at a constant rate by all nucleated cells and importantly is freely filtered in the glomeruli and almost completely reabsorbed and catabolized by the proximal renal tubular cells and serum concentrations are strongly inversely correlated with glomerular filtration rate. However, in addition to representing a more sensitive marker of renal function, an increasing number of studies during the last 10 years has shown that even after adjustment for creatinine, cystatin C represents a strong predictor of cardiovascular risk in the ACS, in patients with manifest atherosclerotic disease, but also in the healthy population (85, 86, 84, 87, 88). There is presently a strong interest from various research groups to further evaluate the prognostic role of cystatin C in large-scale populations.

***Biomarkers of glucose metabolism***

The most eminent markers of glucose metabolism are being represented by fasting and post-prandial glucose, and insulin. In general, there is strong agreement that patients with type 2 diabetes do have an approximately twofold increased risk for cardiovascular outcomes. In addition, several meta-analyses have focused on the linear association between fasting blood sugar and risk of CHD or stroke (89). In contrast to the clear-cut data for diabetes mellitus and related cardiovascular risk in non-diabetics, fasting blood glucose was only moderately and non-linearly associated with vascular complications. This not only applied to vascular mortality but also to cancer mortality, and non-cancer non-cardiovascular mortality (90). There is also evidence from a number of long-term epidemiological studies that increased serum levels of insulin are associated with increased cardiovascular risk. For example in the Busselton Study, baseline insulin was positively associated with 6 year incidence of CHD and mortality from CHD and CVD. However, this increased risk was only seen in men but not in women (91). Similarly, in the Helsinki Policemen Study, one hour and two hour post-load plasma insulin levels both predicted CHD risk even after controlling for an oral glucose tolerance test (92). Finally, in the study of men born in 1913 and 1923 a similar increased risk was seen as in the Busselton Study (92, 93). The simultaneous measurement of fasting glucose and insulin levels offers the opportunity to assess insulin resistance as estimated by the HOMA equation. Insulin resistance is central to the pathophysiology of type 2 diabetes and thus large-scale data in various populations would enhance our understanding of the growing epidemic of diabetes.

***Vitamin D***

An increasing body of evidence suggests that vitamin D deficiency adversely affects the cardiovascular system, but data from longitudinal studies are still scarce (94). Circulating 25-hydroxy vitamin D (25 [OH] D), the most commonly used index of vitamin D status, is converted to the active hormone 1, 25 Dihydroxyvitamin D3 and exerts its physiological effects with the vitamin D receptor (VDR) which is present in various cells of the cardiovascular system including cardiomyocytes, vascular smooth muscle cells, and endothelial cells. Active vitamin D is related to inhibition of cell proliferation, induction of differentiation and apoptosis, improvement of vascular complications, and glycemic control, and thus may protect against cardiovascular disease. Further effects of vitamin D are related to its potential anti-inflammatory properties. More than 50% of the population are considered as vitamin D deficient (95) with a higher prevalence in the older age groups compared to the younger ones which could recently be confirmed in the MONICA/KORA Augsburg Case Cohort Study (96). In elderly, non-institutionalized subjects from Southern Germany the prevalence was 79% (97). Low levels of 25 [OH] D not only seemed to be associated with adverse cardiovascular outcomes but several studies have also related it to diabetes (98), various cancers, and early death (99, 100). While these associations have been reproducibly observed, the question of whether vitamin D is causally associated with cardiovascular disease remains unresolved. The assessment of the prevalence of vitamin D deficiency in various populations from different geographical areas in Europe seems to be of great importance from a public health perspective. Yet ongoing studies still have to determine whether supplementation with vitamin D will reduce adverse outcomes.

**Emerging, literature-based biomarkers**

***Growth-differentiation factor 15 (GDF-15)***

GDF-15 is a distant member of the TGF-β cytokine superfamily. GDF-15 is weakly expressed under healthy conditions but strongly induced upon inflammation and tissue injury. The prognostic value of GDF-15 was first recognized in patients with non-ST-elevation acute coronary syndrome (NSTE-ACS) from the GUSTO-IV and FRISC‑II trials where elevated levels of GDF-15 were associated with an increased risk of mortality independent of clinical risk markers and biomarkers such as CRP and NT-proBNP (101, 102). The independent prognostic value of GDF-15 in NSTE-ACS was recently confirmed in patients from the PLATO trial (103). GDF-15 provides independent prognostic information also in patients with stable angina (as shown in the AtheroGene Study) and chronic heart failure (Val-HeFT trial) (104, 105). Moreover, GDF-15 has been found to be associated with adverse outcomes in population-based cohorts such as the PIVUS, Dallas Heart, Rancho Bernardo, and Framingham Studies (106-110). In these studies, the circulating levels of GDF-15 were independently related to intermediate cardiovascular phenotypes, including endothelial dysfunction, intima media thickness, plaque burden, and left ventricular hypertrophy and dilatation (106, 109). Moreover, GDF-15 emerged as a predictor of all-cause mortality, cardiovascular mortality, non-cardiovascular mortality, cancer mortality, first heart failure events, and worsening kidney function, independent of traditional risk factors and biomarkers such as CRP, NT-proBNP, high-sensitivity cardiac troponin-T, and cystatin-C (107-111). Thus, measurement of GDF-15 may contribute to a refined risk assessment on top of traditional risk factors and biomarkers.

***ST2 (IL-1RL-1, Interleukin 1 receptor-like 1)***

ST2 represents another promising biomarker currently under intensive investigation. It is a member of the interleukin 1 receptor family and its biology involves two distinct and important mechanisms: cardioprotection or conversely remodeling (fibrosis) and hypertrophy and immune response and inflammatory signaling. It has been shown that ST2 levels rise above normal in the context of various cardiac diseases or injury, that it drives cardiac remodeling and/or fibrosis, which occurs in response to myocardial stress and injury such as worsening heart failure and myocardial infarction. Yet it is not just another natriuretic peptide. In contrast to other markers, ST2 is not influenced by various confounding factors such as prior diagnosis of heart failure, body mass index, age, gender, renal function, etc. and, also unlike other markers, levels change in response to specific therapies that improve outcome. Thus, there is a clear-cut interest for the assessment of ST2 levels in diverse populations to further get insight into its potential role as a novel biomarker. ST2 exists in two isoforms; the membrane-bound ST2 ligand (ST2L) and the soluble ST2 which is unbound. The biological effects of IL-33 are transduced by ST2L mitigating cellular responses to mechanical stress. Loss of intact IL-33/ST2 ligand signaling results in unchecked remodeling of ventricular myocardium characterized by excessive myocyte hypertrophy, fibrosis and worsening of left ventricular function along with a higher risk of death from ventricular failure. Favorable responses of the IL-33/ST2L function are thought to be mediated by inhibition of apoptosis and cell death. In contrast to ST2L, soluble ST2 may act as a “decoy” receptor for IL-33 and when present in large enough amounts, ST2 likely interferes significantly with the actions of IL-33 potentially leading to loss of its beneficial effects. Downstream effects of IL-33/ST2L include activation of T helper type 2 (TH2) cells and production of TH2 associated cytokines like IL-5 and IL-13 thus decreasing the TH2 associated inflammatory response. Increased ST2 concentrations are also largely present in other diseases like asthma, sepsis and trauma. Thus, IL-33 may have a cardioprotective role that may be impacted by large amounts of circulating ST2 in cardiovascular disease since capturing of IL-33 by the soluble form of ST2 from the peripheral circulation hampers its beneficial effects. This is of importance as atherosclerosis is a TH1 associated inflammatory disease and a shift towards TH2 is regarded as cardioprotective (112). A few studies so far have assessed the role of soluble ST2 in cardiac patients. ST2 levels in the MERLIN TIMI-36 Trial (113) albeit showing a weak correlation with biomarkers of acute injury and hemodynamic stress were strongly associated with the risk of heart failure after NSTEMI at one year. In addition, there was an increased risk to suffer from recurrent CVD events even after adjustment for various clinical factors, troponin I and BNP. Similarly, in a population of STEMI patients within the CLARITY-TIMI 28 Trial (114) increased levels of soluble ST2 were associated with the consecutive occurrence of heart failure. Recently, the distribution of soluble ST2 and its clinical correlates have been assessed in the Framingham Heart Study. There were clear gender differences, an increase with age and increased levels were associated with diabetes and hypertension (115). Finally, sST2 added prognostic value to traditional risk factors even in combination with GDF-15 and hsTn I in the Framingham study (110). Thus, there seems to be a broad spectrum of patient settings to which this marker may be applied for prognostic purposes and improved risk stratification but also as a potential indicator of response to treatment in particular in those with heart failure (116, 117, 118).

***Galectin-3***

Galectins are a family of soluble β-galactoside binding lectins that play many important regulatory roles in inflammation, immunity and cancer. Recently, a role of galectin-3 in the pathophysiology of heart failure has been suggested (119). Galectin-3 is upregulated in left ventricular hypertrophy, it stimulates macrophage migration and fibroblast proliferation as well as the development of fibrosis. Its effects are being mediated by complex pathways involving various mediators in particular matrix metalloproteinases and TIMPs in the context of fibrosis forming. This observation in particular may be relevant to cardiac remodeling as an accepted important determinant of clinical outcome in heart failure patients. Interestingly, its inhibition may block or reverse this deleterious process. Recent data from the PROVE-IT TIMI-2 Study (120) suggest an additional value of galectin-3 measurement on top of BNP for the prediction of heart failure after ACS. High BNP and high galectin-3 levels were associated with an almost 5-fold increased risk for incident heart failure compared to a 1.5 and 2 fold increased risk if only galectin-3 or BNP were elevated. However, the study was small and findings need to be replicated in larger cohorts. In another study of similar size, patients who were admitted with shortness of breath to the emergency department, BNP and MRproANP were the only independent diagnostic markers of acute heart failure and provided similar diagnostic information (AUC 0.92 and 0.88, respectively) among a large number of emerging biomarkers although galectin-3 had not been tested (121). Data in the general population on the potential value of galectin-3 for heart failure, death and overall cardiovascular events are available from the Framingham Offspring cohort. In more than 3,000 subjects followed for more than 8 years, those being in the top quartile of the galectin-3 distribution had a clearly increased risk for incident heart failure compared to those in the lower three quartiles. This was true even in multivariable adjusted models taking into account not only traditional risk factors and comorbidities but also BNP. A somewhat less strong association was found for total mortality (122). Further data from the large controlled Rosuvastatin Multinational Trial in Heart Failure (CORONA) suggest that patients with systolic heart failure of ischemic origin with galectin-3 values < 90 ng/ml may benefit from rosuvastatin treatment. However, this is based on post-hoc analysis and serves only as hypothesis generating but still it may pave the road for this marker of fibrosis being used to assess response to statin treatment if confirmed in other studies (123).

**Reference List**

1. Rapola JM, Virtamo J, Korhonen P, Haapakoski J, Hartman AM, Edwards BK, Heinonen OP. Validity of diagnoses of major coronary events in national registers of hospital diagnoses and deaths in Finland. Eur J Epidemiol. 1997;13(2):133-8. PMID: 9084994
2. [Leppälä JM](http://www.ncbi.nlm.nih.gov/pubmed?term=Lepp%C3%A4l%C3%A4%20JM%5BAuthor%5D&cauthor=true&cauthor_uid=10484542) Virtamo J, Heinonen OP. Validation of stroke diagnosis in the National Hospital Discharge Register and the Register of Causes of Death in Finland. Eur J Epidemiol 1999;15:155-60.
3. The ATBC Cancer Prevention Study Group. The Alpha-Tocopherol, Beta-Carotene Lung Cancer Prevention Study: design, methods, participant characteristics, and compliance. Ann Epidemiol. 1994;4:1-10. PMID: 8205268
4. Yarnell JWG, Baker IA, Sweetnam PM, Bainton D, O’Brien JR, Whitehead PJ, Elwood PC. Fibrinogen, viscosity and white blood cell count are major risk factors for ischemic heart disease. The Caerphilly and Speedwell collaborative heart disease studies. Circulation 1991, 83:836-844. PMID: 1999035
5. Leitsalu L, Haller T, Esko T, Tammesoo M-L, Alavere H, Snieder H, Perola M, Ng PC, Mägi R, Milani L, Fischer K, Metspalu A. Cohort Profile: Estonian Biobank of the Estonian Genome Center, University of Tartu. Int J. Epidemiol. 2013 Online early
6. Wild P, Sinning CR, Roth A, Wilde S, Schnabel RB, Lubos E, Zeller T, Keller T, LacknerKL, Blettner M, Vasan RS, Munzel T, Blankenberg S. Distribution and categorization of left ventricular measurements in the general population. Results from the population-based Gutenberg Heart Study. Circ Cardiovasc Imaging 2010, 3:604-613. PMID: 20643817
7. [Osler M](http://www.ncbi.nlm.nih.gov/pubmed?term=Osler%20M%5BAuthor%5D&cauthor=true&cauthor_uid=20338891), [Linneberg A](http://www.ncbi.nlm.nih.gov/pubmed?term=Linneberg%20A%5BAuthor%5D&cauthor=true&cauthor_uid=20338891), [Glümer C](http://www.ncbi.nlm.nih.gov/pubmed?term=Gl%C3%BCmer%20C%5BAuthor%5D&cauthor=true&cauthor_uid=20338891), [Jørgensen T](http://www.ncbi.nlm.nih.gov/pubmed?term=J%C3%B8rgensen%20T%5BAuthor%5D&cauthor=true&cauthor_uid=20338891). The cohorts at the Research Centre for Prevention and Health, formerly 'The Glostrup Population Studies'. [Int J Epidemiol.](http://www.ncbi.nlm.nih.gov/pubmed/20338891) 2011 Jun;40(3):602-10 PMID: 20338891
8. [Andersen TF](http://www.ncbi.nlm.nih.gov/pubmed?term=Andersen%20TF%5BAuthor%5D&cauthor=true&cauthor_uid=10421985), [Madsen M](http://www.ncbi.nlm.nih.gov/pubmed?term=Madsen%20M%5BAuthor%5D&cauthor=true&cauthor_uid=10421985), [Jørgensen J](http://www.ncbi.nlm.nih.gov/pubmed?term=J%C3%B8rgensen%20J%5BAuthor%5D&cauthor=true&cauthor_uid=10421985), [Mellemkjoer L](http://www.ncbi.nlm.nih.gov/pubmed?term=Mellemkjoer%20L%5BAuthor%5D&cauthor=true&cauthor_uid=10421985), [Olsen JH](http://www.ncbi.nlm.nih.gov/pubmed?term=Olsen%20JH%5BAuthor%5D&cauthor=true&cauthor_uid=10421985). The Danish National Hospital Register. A valuable source of data for modern health sciences. [Dan Med Bull.](http://www.ncbi.nlm.nih.gov/pubmed/10421985) 1999 Jun;46(3):263-8. PMID: 10421985
9. [Juel K](http://www.ncbi.nlm.nih.gov/pubmed?term=Juel%20K%5BAuthor%5D&cauthor=true&cauthor_uid=10514943), [Helweg-Larsen K](http://www.ncbi.nlm.nih.gov/pubmed?term=Helweg-Larsen%20K%5BAuthor%5D&cauthor=true&cauthor_uid=10514943). The Danish registers of causes of death. [Dan Med Bull.](http://www.ncbi.nlm.nih.gov/pubmed/10514943) 1999 Sep;46(4):354-7. PMID: 10514943
10. Madsen M, Davidsen M, Rasmussen S, Abildstrom SZ, Osler M. The validity of the diagnosis of acute myocardial infarction in routine statistics: a comparison of mortality and hospital discharge data with the Danish MONICA registry. J Clin Epidemiol. 2003 Feb;56(2):124-30. PMID: 12654406
11. Peasey A, Bobak M, Kubinova R, Malyutina S, Pajak A, Tamosiunas A, Pikhart H, Nicholson A, Marmot M. 2006. Determinants of cardiovascular disease and other non-communicable diseases in Central and Eastern Europe: rationale and design of the HAPIEE study. BMC Public Health 2006, 6:255. 2006. PMID: 17049075
12. Löwel H, Lewis M, Hörmann A, Keil U. Case finding, data quality aspects and comparability of myocardial infarction registers: Results of a south German register study. J Clin Epidemiol. 1991;44(3):249-260. PMID: 1999684
13. Meisinger C, Srrassburger K, Heier M, Thorand B, Baumeister SE, Giani G, Rathmann W. Prevalence of undiagnosed diabetes and impaired glucose regulation in 35-59-year-old individuals in Southern Germany: the KORA F4 Study. Diabet Med. 2010 Mar;27(3):360-2. PMID:20536501
14. Santimone I, Di Castelnuovo A, De Curtis A, Spinelli M, Cugino D, Gianfagna F,Zito F, Donati MB, Cerletti C, de Gaetano G, Iacoviello L; MOLI-SANI Project Investigators. White blood cell count, sex and age are major determinants of heterogeneity of platelet indices in an adult general population: results from the MOLI-SANI project. Haematologica. 2011;96:1180-8. PMID: 21546503
15. Ferrario M, Sega R, Chatenoud L, Mancia G, Mocarelli P, Crespi C, Cesana G. Time trends of major coronary risk factors in a northern Italian population (1986-1994). How remarkable are socio-economic differences in an industrialized low CHD incidence country? *Int J Epidemiol* 2001;30:285-291. PMID: 11369728
16. Ferrario M, Chiodini P, Chambless LE, Cesana G, Vanuzzo D, Panico S, Sega R, Pilotto L, Palmieri L, Giampaoli S. for the CUORE Project Research Group. Prediction of coronary events in a low incidence population. Assessing accuracy of the CUORE Cohort Study prediction equation. Int J Epidemiol 2005; 34: 413-21. PMID:15659467
17. Rodés A, Sans S, Balañá Ll, Paluzie G, Aguilera R, Balaguer-Vintró I. Recruitment methods and differences in early, late and non-respondents in the first MONICA-CATALONIA population survey. Rev Epidemiol Sante Publique 1990; 38: 447-454. PMID: 2082450
18. Sans S, Paluzie G, Balan’a L, Puig T, Balaguer-Vintr’o I. Trends in the prevalence, awareness treatment and control of arterial hypertension between 1986 and 1996: the MONICA-Catalonia study. Med Clin 2001;117: 246;253. PMID 11562336
19. [Sans S](http://www.ncbi.nlm.nih.gov/pubmed?term=Sans%20S%5BAuthor%5D&cauthor=true&cauthor_uid=15618037), [Puigdefábregas A](http://www.ncbi.nlm.nih.gov/pubmed?term=Puigdef%C3%A1bregas%20A%5BAuthor%5D&cauthor=true&cauthor_uid=15618037), [Paluzie G](http://www.ncbi.nlm.nih.gov/pubmed?term=Paluzie%20G%5BAuthor%5D&cauthor=true&cauthor_uid=15618037), [Monterde D](http://www.ncbi.nlm.nih.gov/pubmed?term=Monterde%20D%5BAuthor%5D&cauthor=true&cauthor_uid=15618037), [Balaguer-Vintró I](http://www.ncbi.nlm.nih.gov/pubmed?term=Balaguer-Vintr%C3%B3%20I%5BAuthor%5D&cauthor=true&cauthor_uid=15618037). Increasing trends of acute myocardial infarction in Spain: the MONICA-Catalonia Study. [Eur Heart J.](http://www.ncbi.nlm.nih.gov/pubmed/15618037) 2005 Mar;26(5):505-15. PMID: 15618037
20. Eriksson M, Holmgren L, Janlert U, Jansson JH, Lundblad D, Stegmayr B, et al. Large improvements in major cardiovascular risk factors in the population of northern Sweden: the MONICA study 1986-2009. Journal of internal medicine. 2011;269(2):219-31. PMID: 21158982
21. Koster M, Asplund K, Johansson A, Stegmayr B. Refinement of Swedish administrative registers to monitor stroke events on the national level. Neuroepidemiology. 2013;40(4):240-6. PMID: 23364278
22. Ronaldsson O, Norberg M, Nyström L, Söderberg S, Svensson M, Lindahl B, Weinehall L. How to diagnose and classify diabetes in primary health care: lessons learned from the Diabetes Register in Northern Sweden (DiabNorth). Scand J Prim Health Care 2012 Jun;30(2):81-7. PMID: 22643152
23. Yarnell J, The PRIME Study Group; The PRIME Study; classic risk factors do not explain the severalfold differnces in risk of coronary heart disease between France and Northern Ireland. Q J Med 1998, 91:667-676. PMID: 10024924
24. Kendrick S, Clarke J. The Scottish Record Linkage System. Health Bull (Edinb). 1993; 51(2):72–9.
25. Tunstall-Pedoe H, Woodward M, Tavendale R, A’Brook R, McCluskey MK. Comparison of the prediction by 27 different factors of coronary heart disease and death in men and women of the Scottish Heart Health Study: cohort study. BMJ 1997 315(7110):722–9. PMID: 9314758
26. Völzke et al., Int J Epidemiol. 2011 Apr;40(2):294-307. PMID: 20167617
27. Giampaoli S. et al Short term changes of cardiovascular risk factors in the Di.S.Co. Intervention Project. European Journal of Epidemiology 1991, 7: 372-379 PMID: 1915790
28. Jacobsen BK, Eggen AE, Mathiesen EB, Wilsgaard T, Njølstad I. Cohort profile: The Tromsø Study. Int J Epidemiol 2011, 1-7. PMID: 21422063
29. Mannsverk J, Wilsgaard T, Njølstad I, Hopstock LA, Løchen ML, Mathiesen EB, Thelle DS, Bønaa KH. Age and gender differences in incidence and case fatality trends for myocardial infarction: a 30 year follow-up. The Tromsø Study. Eur J Prev Cardiol 2012;19:927-934. PMID: 21859780
30. Mathiesen EB, Johnsen SH, Wilsgaard T, Bønaa KH, Løchen ML, Njølstad I: Carotid plaque area and intima-media thickness in prediction of first-ever ischemic stroke. A 10-year follow-up of 6584 men and women. The Tromsø Study. Stroke 2011;42:972-978. PMID: 21311059
31. [Blankenberg S, Rupprecht HJ, Bickel C](http://www.ncbi.nlm.nih.gov/pubmed?term=Bickel%20C%5BAuthor%5D&cauthor=true&cauthor_uid=14573732), [Torzewski M](http://www.ncbi.nlm.nih.gov/pubmed?term=Torzewski%20M%5BAuthor%5D&cauthor=true&cauthor_uid=14573732), [Hafner G](http://www.ncbi.nlm.nih.gov/pubmed?term=Hafner%20G%5BAuthor%5D&cauthor=true&cauthor_uid=14573732), [Tiret L](http://www.ncbi.nlm.nih.gov/pubmed?term=Tiret%20L%5BAuthor%5D&cauthor=true&cauthor_uid=14573732), [Smieja M](http://www.ncbi.nlm.nih.gov/pubmed?term=Smieja%20M%5BAuthor%5D&cauthor=true&cauthor_uid=14573732), [Cambien F](http://www.ncbi.nlm.nih.gov/pubmed?term=Cambien%20F%5BAuthor%5D&cauthor=true&cauthor_uid=14573732), [Meyer J](http://www.ncbi.nlm.nih.gov/pubmed?term=Meyer%20J%5BAuthor%5D&cauthor=true&cauthor_uid=14573732), [Lackner KJ](http://www.ncbi.nlm.nih.gov/pubmed?term=Lackner%20KJ%5BAuthor%5D&cauthor=true&cauthor_uid=14573732) for the [AtheroGene Investigators](http://www.ncbi.nlm.nih.gov/pubmed?term=AtheroGene%20Investigators%5BCorporate%20Author%5D). Glutathione peroxidase 1 activity and cardiovascular events in patients with coronary artery disease. NEJM; 2003; 349:17:1605-13. PMID: 14573732
32. Reichlin T, Hochholzer W, Bassetti S, Steuer S, Stelzig C, Hartwiger S, Biedert S, Schaub N, Buerge C, Potocki M, Noveanu M, Breidthardt T, Twerenbold R, Winkler K, Bingisser R, Mueller C. Early diagnosis of myocardial infarction with sensitive cardiac troponin assays. N Engl J Med. 2009 Aug 27;361(9):858-67. PMID: 19710484
33. Rothenbacher D, Koenig W, Brenner H. Comparison of N-terminal-pro-B-natriuretic peptide, C-reactive protein, and creatinine clearance for prognosis in patients with known coronary heart disease. Archives of Internal Medicine 2006; 166: 2428-2430. PMID: 17159010
34. Breitling LP, Salzmann K, Rothenbacher D, Burwinkel B, Brenner H. Smoking, F2RL3 methylation and prognosis in stable coronary heart disease. European Heart Journal 2012; 33: 2841-2848. PMID: 22511653
35. Keller T, Zeller T, Ojeda F, Tzikas S, Lillpopp L, Sinning C, Wild P, Genth-Zotz S, Warnholtz A, Giannitsis E, Mockel M, Bickel C, Peetz D, Lackner K, Baldus S, Munzel T, Blankenberg S. Serial changes in highly sensitive troponin I assay and early diagnosis of myocardial infarction. JAMA. 2011 Dec 28;306(24):2684-93. PMID: 22203537
36. Keller T, Zeller T, Peetz D, Tzikas S, Roth A, Czyz E, Bickel C, Baldus S, Warnholtz A, Frohlich M, Sinning CR, Eleftheriadis MS, Wild PS, Schnabel RB, Lubos E, Jachmann N, Genth-Zotz S, Post F, Nicaud V, Tiret L, Lackner KJ, Munzel TF, Blankenberg S. Sensitive troponin I assay in early diagnosis of acute myocardial infarction. N Engl J Med. 2009 Aug 27;361(9):868-77. PMID: 19710485
37. Biasucci LM, Liuzzo G, Della Bona R, Leo M, Biasillo G, Angiolillo DJ, Abbate A, Rizzello V, Niccoli G, Giubilato S, Crea F. Different apparent prognostic value of hsCRP in type 2 diabetic and nondiabetic patients with acute coronary syndromes. Clin Chem. 2009 Feb;55(2):365-8.
38. [Serruys PW](http://www.ncbi.nlm.nih.gov/pubmed?term=Serruys%20PW%5BAuthor%5D&cauthor=true&cauthor_uid=18765397), [García-García HM](http://www.ncbi.nlm.nih.gov/pubmed?term=Garc%C3%ADa-Garc%C3%ADa%20HM%5BAuthor%5D&cauthor=true&cauthor_uid=18765397), [Buszman P](http://www.ncbi.nlm.nih.gov/pubmed?term=Buszman%20P%5BAuthor%5D&cauthor=true&cauthor_uid=18765397), [Erne P](http://www.ncbi.nlm.nih.gov/pubmed?term=Erne%20P%5BAuthor%5D&cauthor=true&cauthor_uid=18765397), [Verheye S](http://www.ncbi.nlm.nih.gov/pubmed?term=Verheye%20S%5BAuthor%5D&cauthor=true&cauthor_uid=18765397), [Aschermann M](http://www.ncbi.nlm.nih.gov/pubmed?term=Aschermann%20M%5BAuthor%5D&cauthor=true&cauthor_uid=18765397), [Duckers H](http://www.ncbi.nlm.nih.gov/pubmed?term=Duckers%20H%5BAuthor%5D&cauthor=true&cauthor_uid=18765397), [Bleie O](http://www.ncbi.nlm.nih.gov/pubmed?term=Bleie%20O%5BAuthor%5D&cauthor=true&cauthor_uid=18765397), [Dudek D](http://www.ncbi.nlm.nih.gov/pubmed?term=Dudek%20D%5BAuthor%5D&cauthor=true&cauthor_uid=18765397), [Bøtker HE](http://www.ncbi.nlm.nih.gov/pubmed?term=B%C3%B8tker%20HE%5BAuthor%5D&cauthor=true&cauthor_uid=18765397), [von Birgelen C](http://www.ncbi.nlm.nih.gov/pubmed?term=von%20Birgelen%20C%5BAuthor%5D&cauthor=true&cauthor_uid=18765397), [D'Amico D](http://www.ncbi.nlm.nih.gov/pubmed?term=D'Amico%20D%5BAuthor%5D&cauthor=true&cauthor_uid=18765397), [Hutchinson T](http://www.ncbi.nlm.nih.gov/pubmed?term=Hutchinson%20T%5BAuthor%5D&cauthor=true&cauthor_uid=18765397), [Zambanini A](http://www.ncbi.nlm.nih.gov/pubmed?term=Zambanini%20A%5BAuthor%5D&cauthor=true&cauthor_uid=18765397), [Mastik F](http://www.ncbi.nlm.nih.gov/pubmed?term=Mastik%20F%5BAuthor%5D&cauthor=true&cauthor_uid=18765397), [van Es GA](http://www.ncbi.nlm.nih.gov/pubmed?term=van%20Es%20GA%5BAuthor%5D&cauthor=true&cauthor_uid=18765397), [van der Steen AF](http://www.ncbi.nlm.nih.gov/pubmed?term=van%20der%20Steen%20AF%5BAuthor%5D&cauthor=true&cauthor_uid=18765397), [Vince DG](http://www.ncbi.nlm.nih.gov/pubmed?term=Vince%20DG%5BAuthor%5D&cauthor=true&cauthor_uid=18765397), [Ganz P](http://www.ncbi.nlm.nih.gov/pubmed?term=Ganz%20P%5BAuthor%5D&cauthor=true&cauthor_uid=18765397), [Hamm CW](http://www.ncbi.nlm.nih.gov/pubmed?term=Hamm%20CW%5BAuthor%5D&cauthor=true&cauthor_uid=18765397), [Wijns W](http://www.ncbi.nlm.nih.gov/pubmed?term=Wijns%20W%5BAuthor%5D&cauthor=true&cauthor_uid=18765397), [Zalewski A](http://www.ncbi.nlm.nih.gov/pubmed?term=Zalewski%20A%5BAuthor%5D&cauthor=true&cauthor_uid=18765397); [Integrated Biomarker and Imaging Study-2 Investigators](http://www.ncbi.nlm.nih.gov/pubmed?term=Integrated%20Biomarker%20and%20Imaging%20Study-2%20Investigators%5BCorporate%20Author%5D). Effects of the direct lipoprotein-associated phospholipase A(2) inhibitor darapladib on human coronary atherosclerotic plaque. [Circulation.](http://www.ncbi.nlm.nih.gov/pubmed/18765397) 2008 Sep 9;118(11):1172-82. PMID: 18765397
39. The Long-Term Intervention with Pravastatin in Ischaemic Disease (LIPID) Study Group. Prevention of cardiovascular events and death with pravastatin in patients with coronary heart disease and a broad range of initial cholesterol levels. N [Engl J Med.](http://www.ncbi.nlm.nih.gov/pubmed/9841303) 1998 Nov 5;339(19):1349-57. PMID: 9841303
40. Ridker PM; JUPITER Study Group. Rosuvastatin in the primary prevention of cardiovascular disease among patients with low levels of low-density lipoprotein cholesterol and elevated high-sensitivity C-reactive protein: rationale and design of JUPITER trial. Circulation 2003:108;19:2292-7.
41. 41. [Ridker PM](http://www.ncbi.nlm.nih.gov/pubmed?term=Ridker%20PM%5BAuthor%5D&cauthor=true&cauthor_uid=18997196), [Danielson E](http://www.ncbi.nlm.nih.gov/pubmed?term=Danielson%20E%5BAuthor%5D&cauthor=true&cauthor_uid=18997196), [Fonseca FA](http://www.ncbi.nlm.nih.gov/pubmed?term=Fonseca%20FA%5BAuthor%5D&cauthor=true&cauthor_uid=18997196), [Genest J](http://www.ncbi.nlm.nih.gov/pubmed?term=Genest%20J%5BAuthor%5D&cauthor=true&cauthor_uid=18997196), [Gotto AM Jr](http://www.ncbi.nlm.nih.gov/pubmed?term=Gotto%20AM%20Jr%5BAuthor%5D&cauthor=true&cauthor_uid=18997196), [Kastelein JJ](http://www.ncbi.nlm.nih.gov/pubmed?term=Kastelein%20JJ%5BAuthor%5D&cauthor=true&cauthor_uid=18997196), [Koenig W](http://www.ncbi.nlm.nih.gov/pubmed?term=Koenig%20W%5BAuthor%5D&cauthor=true&cauthor_uid=18997196), [Libby P](http://www.ncbi.nlm.nih.gov/pubmed?term=Libby%20P%5BAuthor%5D&cauthor=true&cauthor_uid=18997196), [Lorenzatti AJ](http://www.ncbi.nlm.nih.gov/pubmed?term=Lorenzatti%20AJ%5BAuthor%5D&cauthor=true&cauthor_uid=18997196), [MacFadyen JG](http://www.ncbi.nlm.nih.gov/pubmed?term=MacFadyen%20JG%5BAuthor%5D&cauthor=true&cauthor_uid=18997196), [Nordestgaard BG](http://www.ncbi.nlm.nih.gov/pubmed?term=Nordestgaard%20BG%5BAuthor%5D&cauthor=true&cauthor_uid=18997196), [Shepherd J](http://www.ncbi.nlm.nih.gov/pubmed?term=Shepherd%20J%5BAuthor%5D&cauthor=true&cauthor_uid=18997196), [Willerson JT](http://www.ncbi.nlm.nih.gov/pubmed?term=Willerson%20JT%5BAuthor%5D&cauthor=true&cauthor_uid=18997196), [Glynn RJ](http://www.ncbi.nlm.nih.gov/pubmed?term=Glynn%20RJ%5BAuthor%5D&cauthor=true&cauthor_uid=18997196); [JUPITER Study Group](http://www.ncbi.nlm.nih.gov/pubmed?term=JUPITER%20Study%20Group%5BCorporate%20Author%5D). Rosuvastatin to prevent vascular events in men and women with elevated C-reactive protein. N Engl J Med 2008, 359: 21: 2195-207.
42. [Yusuf S](http://www.ncbi.nlm.nih.gov/pubmed?term=Yusuf%20S%5BAuthor%5D&cauthor=true&cauthor_uid=18757085), [Teo K](http://www.ncbi.nlm.nih.gov/pubmed?term=Teo%20K%5BAuthor%5D&cauthor=true&cauthor_uid=18757085), [Anderson C](http://www.ncbi.nlm.nih.gov/pubmed?term=Anderson%20C%5BAuthor%5D&cauthor=true&cauthor_uid=18757085), [Pogue J](http://www.ncbi.nlm.nih.gov/pubmed?term=Pogue%20J%5BAuthor%5D&cauthor=true&cauthor_uid=18757085), [Dyal L](http://www.ncbi.nlm.nih.gov/pubmed?term=Dyal%20L%5BAuthor%5D&cauthor=true&cauthor_uid=18757085), [Copland I](http://www.ncbi.nlm.nih.gov/pubmed?term=Copland%20I%5BAuthor%5D&cauthor=true&cauthor_uid=18757085), [Schumacher H](http://www.ncbi.nlm.nih.gov/pubmed?term=Schumacher%20H%5BAuthor%5D&cauthor=true&cauthor_uid=18757085), [Dagenais G](http://www.ncbi.nlm.nih.gov/pubmed?term=Dagenais%20G%5BAuthor%5D&cauthor=true&cauthor_uid=18757085), [Sleight P](http://www.ncbi.nlm.nih.gov/pubmed?term=Sleight%20P%5BAuthor%5D&cauthor=true&cauthor_uid=18757085). The Telmisartan Randomised AssessmeNt Study in ACE iNtolerant subjects with cardiovascular Disease (TRANSCEND) Investigators. Effects of the angiotensin-receptor blocker telmisartan on cardiovascular events in high-risk patients intolerant to angiotensin-converting enzyme inhibitors; a randomised controlled trial. The Lancet 2008, 27;372(9644):1174-83 PMID: 18757085
43. Ridker PM, Cook NR, Lee IM, Gordon D, Gaziano JM, Manson JE, Hennekens CH, Buring JE. A randomised trial of low-dose aspirin in the primary prevention of cardiovascular disease in women. NEJM, 2005;352:13:1293-304 PMID: 15753114
44. The Women’s Health Initiative Study Group. Design of the Women’s Health Initiative Clinical Trial and Observational Study. Controlled Clinical Trials; 1998:19:1:61-109. PMID: 9492970
45. Zeller T, Tunstall-Pedoe H, Saarela O, Ojeda F, Schnabel RB, Tuovinen T, Woodward M, Struthers A, Hughes M, Kee F, Salomaa V, Kuulasmaa K, Blankenberg S for the MORGAM Investigators. High Population Prevalence of Cardiac Troponin I measured by a high-sensitivity assay and Cardiovascular Risk Estimation. The MORGAM Biomarker project Scottish cohort. Eur Heart J. 2013; 8. PMID: 24104876
46. Thygesen K, Mair J, Katus H, Plebani M, Venge P, Collinson P, et al. Recommendations for the use of cardiac troponin measurement in acute cardiac care. *Eur Heart J* 2010 Sep;31(18):2197-2204.
47. Thygesen K, Mair J, Giannitsis E, Mueller C, Lindahl B, Blankenberg S, et al. How to use high-sensitivity cardiac troponins in acute cardiac care. *Eur Heart J* 2012 Sep;33(18):2252-2257.
48. Apple FS, Collinson PO, IFCC Task Force on Clinical Applications of Cardiac Biomarkers. Analytical characteristics of high-sensitivity cardiac troponin assays. *Clin Chem* 2012 Jan;58(1):54-61.
49. Mueller M, Biener M, Vafaie M, Doerr S, Keller T, Blankenberg S, et al. Absolute and relative kinetic changes of high-sensitivity cardiac troponin T in acute coronary syndrome and in patients with increased troponin in the absence of acute coronary syndrome. *Clin Chem* 2012 Jan;58(1):209-218.
50. Omland T, de Lemos JA, Sabatine MS, Christophi CA, Rice MM, Jablonski KA, et al. A sensitive cardiac troponin T assay in stable coronary artery disease. *N Engl J Med* 2009 Dec 24;361(26):2538-2547.
51. Koenig W, Breitling LP, Hahmann H, Wusten B, Brenner H, Rothenbacher D. Cardiac troponin T measured by a high-sensitivity assay predicts recurrent cardiovascular events in stable coronary heart disease patients with 8-year follow-up. *Clin Chem* 2012 Aug;58(8):1215-1224.
52. Kistorp C, Raymond I, Pedersen F, Gustafsson F, Faber J, Hildebrandt P. N-terminal pro-brain natriuretic peptide, C-reactive protein, and urinary albumin levels as predictors of mortality and cardiovascular events in older adults. *JAMA* 2005 Apr 6;293(13):1609-1616.
53. Blankenberg S, McQueen MJ, Smieja M, Pogue J, Balion C, Lonn E, et al. Comparative impact of multiple biomarkers and N-Terminal pro-brain natriuretic peptide in the context of conventional risk factors for the prediction of recurrent cardiovascular events in the Heart Outcomes Prevention Evaluation (HOPE) Study. *Circulation* 2006 Jul 18;114(3):201-208.
54. Morrow DA. Cardiovascular risk prediction in patients with stable and unstable coronary heart disease. *Circulation* 2010 Jun 22;121(24):2681-2691.
55. Di AE, Chowdhury R, Sarwar N, Ray KK, Gobin R, Saleheen D, et al. B-type natriuretic peptides and cardiovascular risk: systematic review and meta-analysis of 40 prospective studies. *Circulation* 2009 Dec 1;120(22):2177-2187.
56. Fard A, Maisel A. Natriuretic peptides, novel biomarkers, and the prediction of future events. *Eur Heart J* 2013 Feb;34(6):419-421.
57. Welsh P, Doolin O, Willeit P, Packard C, Macfarlane P, Cobbe S, et al. N-terminal pro-B-type natriuretic peptide and the prediction of primary cardiovascular events: results from 15-year follow-up of WOSCOPS. *Eur Heart J* 2013 Feb;34(6):443-450.
58. Schou M, Gustafsson F, Videbaek L, Tuxen C, Keller N, Handberg J, et al. Extended heart failure clinic follow-up in low-risk patients: a randomized clinical trial (NorthStar). *Eur Heart J* 2013 Feb;34(6):432-442.
59. Thygesen K, Mair J, Mueller C, Huber K, Weber M, Plebani M, et al. Recommendations for the use of natriuretic peptides in acute cardiac care: a position statement from the Study Group on Biomarkers in Cardiology of the ESC Working Group on Acute Cardiac Care. *Eur Heart J* 2012 Aug;33(16):2001-2006.
60. Emerging Risk FC, Kaptoge S, Di AE, Lowe G, Pepys MB, Thompson SG, et al. C-reactive protein concentration and risk of coronary heart disease, stroke, and mortality: an individual participant meta-analysis. *Lancet* 2010 Jan 9;375(9709):132-140.
61. Emerging Risk FC, Kaptoge S, Di AE, Pennells L, Wood AM, White IR, et al. C-reactive protein, fibrinogen, and cardiovascular disease prediction. *N Engl J Med* 2012 Oct 4;367(14):1310-1320.
62. Greenland P, Alpert JS, Beller GA, Benjamin EJ, Budoff MJ, Fayad ZA, et al. 2010 ACCF/AHA guideline for assessment of cardiovascular risk in asymptomatic adults: a report of the American College of Cardiology Foundation/American Heart Association Task Force on Practice Guidelines. *J Am Coll Cardiol* 2010 Dec 14;56(25):e50-103.
63. Buckley DI, Fu R, Freeman M, Rogers K, Helfand M. C-reactive protein as a risk factor for coronary heart disease: a systematic review and meta-analyses for the U.S. Preventive Services Task Force. *Ann Intern Med* 2009 Oct 6;151(7):483-495.
64. Genest J, McPherson R, Frohlich J, Anderson T, Campbell N, Carpentier A, et al. 2009 Canadian Cardiovascular Society/Canadian guidelines for the diagnosis and treatment of dyslipidemia and prevention of cardiovascular disease in the adult - 2009 recommendations. *Can J Cardiol* 2009 Oct;25(10):567-579.
65. Ridker PM, Danielson E, Fonseca FA, Genest J, Gotto AM, Jr., Kastelein JJ, et al. Rosuvastatin to prevent vascular events in men and women with elevated C-reactive protein. *N Engl J Med* 2008 Nov 20;359(21):2195-2207.
66. Sondermeijer BM, Boekholdt SM, Rana JS, Kastelein JJ, Wareham NJ, Khaw KT. Clinical implications of JUPITER in a contemporary European population: the EPIC-Norfolk prospective population study. *Eur Heart J* 2013 Feb 28.
67. European Association for Cardiovascular Prevention & Rehabilitation, Reiner Z, Catapano AL, De BG, Graham I, Taskinen MR, et al. ESC/EAS Guidelines for the management of dyslipidaemias: the Task Force for the management of dyslipidaemias of the European Society of Cardiology (ESC) and the European Atherosclerosis Society (EAS). *Eur Heart J* 2011 Jul;32(14):1769-1818.
68. Perk J, De BG, Gohlke H, Graham I, Reiner Z, Verschuren M, et al. European Guidelines on cardiovascular disease prevention in clinical practice (version 2012). The Fifth Joint Task Force of the European Society of Cardiology and Other Societies on Cardiovascular Disease Prevention in Clinical Practice (constituted by representatives of nine societies and by invited experts). *Eur Heart J* 2012 Jul;33(13):1635-1701.
69. Emerging Risk FC, Di AE, Gao P, Pennells L, Kaptoge S, Caslake M, et al. Lipid-related markers and cardiovascular disease prediction. *JAMA* 2012 Jun 20;307(23):2499-2506.
70. Emerging Risk FC, Di AE, Sarwar N, Perry P, Kaptoge S, Ray KK, et al. Major lipids, apolipoproteins, and risk of vascular disease. *JAMA* 2009 Nov 11;302(18):1993-2000.
71. Langsted A, Freiberg JJ, Tybjaerg-Hansen A, Schnohr P, Jensen GB, Nordestgaard BG. Nonfasting cholesterol and triglycerides and association with risk of myocardial infarction and total mortality: the Copenhagen City Heart Study with 31 years of follow-up. *J Intern Med* 2011 Jul;270(1):65-75.
72. Varbo A, Nordestgaard BG, Tybjaerg-Hansen A, Schnohr P, Jensen GB, Benn M. Nonfasting triglycerides, cholesterol, and ischemic stroke in the general population. *Ann Neurol* 2011 Apr;69(4):628-634.
73. Nordestgaard BG, Langsted A, Freiberg JJ. Nonfasting hyperlipidemia and cardiovascular disease. *Curr Drug Targets* 2009 Apr;10(4):328-335.
74. Triglyceride Coronary Disease Genetics Consortium and Emerging Risk Factors, Sarwar N, Sandhu MS, Ricketts SL, Butterworth AS, Di AE, et al. Triglyceride-mediated pathways and coronary disease: collaborative analysis of 101 studies. *Lancet* 2010 May 8;375(9726):1634-1639.
75. Castelli WP, Garrison RJ, Wilson PW, Abbott RD, Kalousdian S, Kannel WB. Incidence of coronary heart disease and lipoprotein cholesterol levels. The Framingham Study. *JAMA* 1986 Nov 28;256(20):2835-2838.
76. Bayturan O, Kapadia S, Nicholls SJ, Tuzcu EM, Shao M, Uno K, et al. Clinical predictors of plaque progression despite very low levels of low-density lipoprotein cholesterol. *J Am Coll Cardiol* 2010 Jun 15;55(24):2736-2742.
77. AIM-HIGH Investigators, Boden WE, Probstfield JL, Anderson T, Chaitman BR, svignes-Nickens P, et al. Niacin in patients with low HDL cholesterol levels receiving intensive statin therapy. *N Engl J Med* 2011 Dec 15;365(24):2255-2267.
78. Armitage J. Niacin causes serious unexpected side-effects, but no worthwhile benefits, for patients who are at increased risk of heart attacks and strokes. http://www.thrivestudy.org/press_release.thm. 2013 March.
79. Chapman MJ, Ginsberg HN, Amarenco P, Andreotti F, Boren J, Catapano AL, et al. Triglyceride-rich lipoproteins and high-density lipoprotein cholesterol in patients at high risk of cardiovascular disease: evidence and guidance for management. *Eur Heart J* 2011 Jun;32(11):1345-1361.
80. Clarke R, Peden JF, Hopewell JC, Kyriakou T, Goel A, Heath SC, et al. Genetic variants associated with Lp(a) lipoprotein level and coronary disease. *N Engl J Med* 2009 Dec 24;361(26):2518-2528.
81. Kamstrup PR, Tybjaerg-Hansen A, Steffensen R, Nordestgaard BG. Genetically elevated lipoprotein(a) and increased risk of myocardial infarction. *JAMA* 2009 Jun 10;301(22):2331-2339.
82. Emerging Risk FC, Erqou S, Kaptoge S, Perry PL, Di AE, Thompson A, et al. Lipoprotein(a) concentration and the risk of coronary heart disease, stroke, and nonvascular mortality. *JAMA* 2009 Jul 22;302(4):412-423.
83. Nordestgaard BG, Chapman MJ, Ray K, Boren J, Andreotti F, Watts GF, et al. Lipoprotein(a) as a cardiovascular risk factor: current status. *Eur Heart J* 2010 Dec;31(23):2844-2853.
84. Zhang QL, Brenner H, Koenig W, Rothenbacher D. Prognostic value of chronic kidney disease in patients with coronary heart disease: role of estimating equations. *Atherosclerosis* 2010 Jul;211(1):342-347.
85. Jernberg T, Lindahl B, James S, Larsson A, Hansson LO, Wallentin L. Cystatin C: a novel predictor of outcome in suspected or confirmed non-ST-elevation acute coronary syndrome. *Circulation* 2004 Oct 19;110(16):2342-2348.
86. Koenig W, Twardella D, Brenner H, Rothenbacher D. Plasma concentrations of cystatin C in patients with coronary heart disease and risk for secondary cardiovascular events: more than simply a marker of glomerular filtration rate. *Clin Chem* 2005 Feb;51(2):321-327.
87. Shlipak MG, Katz R, Sarnak MJ, Fried LF, Newman AB, Stehman-Breen C, et al. Cystatin C and prognosis for cardiovascular and kidney outcomes in elderly persons without chronic kidney disease. *Ann Intern Med* 2006 Aug 15;145(4):237-246.
88. Taglieri N, Koenig W, Kaski JC. Cystatin C and cardiovascular risk. *Clin Chem* 2009 Nov;55(11):1932-1943.
89. Emerging Risk FC, Sarwar N, Gao P, Seshasai SR, Gobin R, Kaptoge S, et al. Diabetes mellitus, fasting blood glucose concentration, and risk of vascular disease: a collaborative meta-analysis of 102 prospective studies. *Lancet* 2010 Jun 26;375(9733):2215-2222.
90. Emerging Risk FC, Seshasai SR, Kaptoge S, Thompson A, Di AE, Gao P, et al. Diabetes mellitus, fasting glucose, and risk of cause-specific death. *N Engl J Med* 2011 Mar 3;364(9):829-841.
91. Welborn TA, Wearne K. Coronary heart disease incidence and cardiovascular mortality in Busselton with reference to glucose and insulin concentrations. *Diabetes Care* 1979 Mar;2(2):154-160.
92. Pyorala K. Relationship of glucose tolerance and plasma insulin to the incidence of coronary heart disease: results from two population studies in Finland. *Diabetes Care* 1979 Mar;2(2):131-141.
93. Welin L, Bresater LE, Eriksson H, Hansson PO, Welin C, Rosengren A. Insulin resistance and other risk factors for coronary heart disease in elderly men. The Study of Men Born in 1913 and 1923. *Eur J Cardiovasc Prev Rehabil* 2003 Aug;10(4):283-288.
94. Judd SE, Tangpricha V. Vitamin D deficiency and risk for cardiovascular disease. *Am J Med Sci* 2009 Jul;338(1):40-44.
95. Holick MF, Binkley NC, Bischoff-Ferrari HA, Gordon CM, Hanley DA, Heaney RP, et al. Evaluation, treatment, and prevention of vitamin D deficiency: an Endocrine Society clinical practice guideline. *J Clin Endocrinol Metab* 2011 Jul;96(7):1911-1930.
96. Karakas M, Thorand B, Zierer A, Huth C, Meisinger C, Roden M, et al. Low levels of serum 25-hydroxyvitamin D are associated with increased risk of myocardial infarction, especially in women: results from the MONICA/KORA Augsburg case-cohort study. *J Clin Endocrinol Metab* 2013 Jan;98(1):272-280.
97. Klenk J, Rapp K, Denkinger MD, Nagel G, Nikolaus T, Peter R, et al. Seasonality of vitamin D status in older people in Southern Germany: implications for assessment. *Age Ageing* 2013 Mar 29.
98. Afzal S, Bojesen SE, Nordestgaard BG. Low 25-hydroxyvitamin D and risk of type 2 diabetes: a prospective cohort study and metaanalysis. *Clin Chem* 2013 Feb;59(2):381-391.
99. Brondum-Jacobsen P, Benn M, Jensen GB, Nordestgaard BG. 25-hydroxyvitamin d levels and risk of ischemic heart disease, myocardial infarction, and early death: population-based study and meta-analyses of 18 and 17 studies. *Arterioscler Thromb Vasc Biol* 2012 Nov;32(11):2794-2802.

100.Thomas GN, Hartaigh B, Bosch JA, Pilz S, Loerbroks A, Kleber ME, et al. Vitamin D levels predict all-cause and cardiovascular disease mortality in subjects with the metabolic syndrome: the Ludwigshafen Risk and Cardiovascular Health (LURIC) Study. *Diabetes Care* 2012 May;35(5):1158-1164.

101. Wollert KC, Kempf T, Peter T, Olofsson S, James S, Johnston N, et al. Prognostic value of growth-differentiation factor-15 in patients with non-ST-elevation acute coronary syndrome. *Circulation* 2007 Feb 27;115(8):962-971.

102.Wollert KC, Kempf T, Lagerqvist B, Lindahl B, Olofsson S, Allhoff T, et al. Growth differentiation factor 15 for risk stratification and selection of an invasive treatment strategy in non ST-elevation acute coronary syndrome. *Circulation* 2007 Oct 2;116(14):1540-1548.

103. Wallentin L, Lindholm D, Siegbahn A, Wernroth L, Becker RC, Cannon CP, Cornel JH, Himmelmann A, Giannitsis E, Harrington RA, Held C, Husted S, Katus HA, Mahaffey KW, Steg PG, Storey RF, James SK; for the PLATO study group. Biomarkers in Relation to the Effects of Ticagrelor Compared with Clopidogrel in Non-ST-Elevation Acute Coronary Syndrome Patients Managed with or without In-Hospital Revascularization: A Substudy from the Prospective Randomized Platelet Inhibition and Patient Outcomes (PLATO) Trial. Circulation 2013 [Epub ahead of print].

104. Kempf T, Sinning JM, Quint A, Bickel C, Sinning C, Wild PS, et al. Growth-differentiation factor-15 for risk stratification in patients with stable and unstable coronary heart disease: results from the AtheroGene study. *Circ Cardiovasc Genet* 2009 Jun;2(3):286-292.

105. Anand IS, Kempf T, Rector TS, Tapken H, Allhoff T, Jantzen F, Kuskowski M, Cohn JN, Drexler H, Wollert KC. Serial measurement of growth-differentiation factor-15 in heart failure: relation to disease severity and prognosis in the Valsartan Heart Failure Trial. *Circulation* 2010 Oct 5;122(14):1387-1395.

106. Lind L, Wallentin L, Kempf T, Tapken H, Quint A, Lindahl B, Olofsson S, Venge P, Larsson A, Hulthe J, Elmgren A, Wollert KC. Growth-differentiation factor-15 is an independent marker of cardiovascular dysfunction and disease in the elderly: results from the Prospective Investigation of the Vasculature in Uppsala Seniors (PIVUS) Study. *Eur Heart J* 2009 Oct;30(19):2346-2353.

107. Wallentin L, Zethelius B, Berglund L, Eggers KM, Lind L, Lindahl B, Wollert KC, Siegbahn A. GDF-15 for prognostication of cardiovascular and cancer morbidity and mortality in men. *PLoS ONE* 2013 Dec 2;8:e78797.

108. Daniels LB, Clopton P, Laughlin GA, Maisel AS, Barrett-Connor E. Growth-differentiation factor-15 is a robust, independent predictor of 11-year mortality risk in community-dwelling older adults: the Rancho Bernardo Study. *Circulation* 2011 May 17;123(19):2101-2110.

109. Rohatgi A, Patel P, Das SR, Ayers CR, Khera A, Martinez-Rumayor A, et al. Association of growth differentiation factor-15 with coronary atherosclerosis and mortality in a young, multiethnic population: observations from the Dallas Heart Study. *Clin Chem* 2012 Jan;58(1):172-182.

110. Wang TJ, Wollert KC, Larson MG, Coglianese E, McCabe EL, Cheng S, et al. Prognostic utility of novel biomarkers of cardiovascular stress: the Framingham Heart Study. *Circulation* 2012 Sep 25;126(13):1596-1604.

111. Ho JE, Hwang SJ, Wollert KC, Larson MG, Cheng S, Kempf T, Vasan RS, Januzzi JL, Wang TJ, Fox CS. Biomarkers of cardiovascular stress and incident chronic kidney disease. *Clin Chem* 2013 Nov;59(11):1613-1620.

112. Willems S, Hoefer I, Pasterkamp G. The role of the interleukin 1 receptor-like 1 (ST2) and Interleukin-33 pathway in cardiovascular disease and cardiovascular risk assessment. *Minerva Med* 2012 Dec;103(6):513-524.

113. Kohli P, Bonaca MP, Kakkar R, Kudinova AY, Scirica BM, Sabatine MS, et al. Role of ST2 in non-ST-elevation acute coronary syndrome in the MERLIN-TIMI 36 trial. *Clin Chem* 2012 Jan;58(1):257-266.

114. Sabatine MS, Morrow DA, Higgins LJ, MacGillivray C, Guo W, Bode C, et al. Complementary roles for biomarkers of biomechanical strain ST2 and N-terminal prohormone B-type natriuretic peptide in patients with ST-elevation myocardial infarction. *Circulation* 2008 Apr 15;117(15):1936-1944.

115. Coglianese EE, Larson MG, Vasan RS, Ho JE, Ghorbani A, McCabe EL, et al. Distribution and clinical correlates of the interleukin receptor family member soluble ST2 in the Framingham Heart Study. *Clin Chem* 2012 Dec;58(12):1673-1681.

116. Januzzi JL, Jr., Peacock WF, Maisel AS, Chae CU, Jesse RL, Baggish AL, et al. Measurement of the interleukin family member ST2 in patients with acute dyspnea: results from the PRIDE (Pro-Brain Natriuretic Peptide Investigation of Dyspnea in the Emergency Department) study. *J Am Coll Cardiol* 2007 Aug 14;50(7):607-613.

117. Rehman SU, Martinez-Rumayor A, Mueller T, Januzzi JL, Jr. Independent and incremental prognostic value of multimarker testing in acute dyspnea: results from the ProBNP Investigation of Dyspnea in the Emergency Department (PRIDE) study. *Clin Chim Acta* 2008 Jun;392(1-2):41-45.

118. Januzzi JL, Jr., Rehman S, Mueller T, van Kimmenade RR, Lloyd-Jones DM. Importance of biomarkers for long-term mortality prediction in acutely dyspneic patients. *Clin Chem* 2010 Dec;56(12):1814-1821.

119. de Boer RA, Voors AA, Muntendam P, van Gilst WH, van Veldhuisen DJ. Galectin-3: a novel mediator of heart failure development and progression. *Eur J Heart Fail* 2009 Sep;11(9):811-817.

120. Grandin EW, Jarolim P, Murphy SA, Ritterova L, Cannon CP, Braunwald E, et al. Galectin-3 and the development of heart failure after acute coronary syndrome: pilot experience from PROVE IT-TIMI 22. *Clin Chem* 2012 Jan;58(1):267-273.

121. Dieplinger B, Gegenhuber A, Haltmayer M, Mueller T. Evaluation of novel biomarkers for the diagnosis of acute destabilised heart failure in patients with shortness of breath. *Heart* 2009 Sep;95(18):1508-1513.

122. Ho JE, Liu C, Lyass A, Courchesne P, Pencina MJ, Vasan RS, et al. Galectin-3, a marker of cardiac fibrosis, predicts incident heart failure in the community. *J Am Coll Cardiol* 2012 Oct 2;60(14):1249-1256.

123. Gullestad L, Ueland T, Kjekshus J, Nymo SH, Hulthe J, Muntendam P, et al. Galectin-3 predicts response to statin therapy in the Controlled Rosuvastatin Multinational Trial in Heart Failure (CORONA). *Eur Heart J* 2012 Sep;33(18):2290-2296.
